# A novel GLM-based method for the Automatic IDentification of functional Events (AIDE) in fNIRS data recorded in naturalistic environments

**DOI:** 10.1016/j.neuroimage.2017.05.001

**Published:** 2017-07-15

**Authors:** Paola Pinti, Arcangelo Merla, Clarisse Aichelburg, Frida Lind, Sarah Power, Elizabeth Swingler, Antonia Hamilton, Sam Gilbert, Paul W. Burgess, Ilias Tachtsidis

**Affiliations:** a Infrared Imaging Lab, Institute for Advanced Biomedical Technology (ITAB), Department of Neuroscience, Imaging and Clinical Sciences, University of Chieti-Pescara, Italy; b Department of Medical Physics and Biomedical Engineering, University College London, UK; c Institute of Cognitive Neuroscience, University College London, UK

**Keywords:** Functional Near Infrared Spectroscopy, General linear model, Functional events, real-world neuroimaging, Onsets identification

## Abstract

Recent technological advances have allowed the development of portable functional Near-Infrared Spectroscopy (fNIRS) devices that can be used to perform neuroimaging in the real-world. However, as real-world experiments are designed to mimic everyday life situations, the identification of event onsets can be extremely challenging and time-consuming. Here, we present a novel analysis method based on the general linear model (GLM) least square fit analysis for the Automatic IDentification of functional Events (or AIDE) directly from real-world fNIRS neuroimaging data. In order to investigate the accuracy and feasibility of this method, as a proof-of-principle we applied the algorithm to (i) synthetic fNIRS data simulating both block-, event-related and mixed-design experiments and (ii) experimental fNIRS data recorded during a conventional lab-based task (involving maths). AIDE was able to recover functional events from simulated fNIRS data with an accuracy of 89%, 97% and 91% for the simulated block-, event-related and mixed-design experiments respectively. For the lab-based experiment, AIDE recovered more than the 66.7% of the functional events from the fNIRS experimental measured data. To illustrate the strength of this method, we then applied AIDE to fNIRS data recorded by a wearable system on one participant during a complex real-world prospective memory experiment conducted outside the lab. As part of the experiment, there were four and six events (actions where participants had to interact with a target) for the two different conditions respectively (condition 1: social-interact with a person; condition 2: non-social-interact with an object). AIDE managed to recover 3/4 events and 3/6 events for conditions 1 and 2 respectively. The identified functional events were then corresponded to behavioural data from the video recordings of the movements and actions of the participant. Our results suggest that “brain-first” rather than “behaviour-first” analysis is possible and that the present method can provide a novel solution to analyse real-world fNIRS data, filling the gap between real-life testing and functional neuroimaging.

## Highlights

• A novel GLM-based method to recover the timing of functional events from fNIRS data.
• The method showed high specificity and sensitivity in synthetic and real data.
• The method is applied to fNIRS data conducted outdoor on a freely moving participant.

## Introduction

Functional Near Infrared Spectroscopy (fNIRS) is a neuroimaging technique able to measure concentration changes in oxygenated (HbO_2_) and deoxygenated (HHb) haemoglobin secondary to neuronal activation. Like functional Magnetic Resonance Imaging (fMRI), fNIRS is a neurovascular coupling-based neuroimaging technique that recovers the hemodynamic response related to functional brain activity. While fMRI relies on the paramagnetic nature of HHb to measure the blood oxygen level-dependent (BOLD) response, fNIRS optically detects changes in HbO_2_ and HHb taking advantage of the low absorption of the biological tissue in the near-infrared range (700–1000 nm) (see Scholkmann et al. (2014a) for a review). Whilst fNIRS is a relatively new neuroimaging method, over the last 20 years it has become a popular tool for clinical and psychological applications (Boas et al., 2014), being extensively used to monitor brain activity in response to a wide variety of cognitive tasks. The fast spreading of this technology is also related to the advantages that the technique offers. For example, thanks to its being non-invasive, portable and robust to motion artifacts, fNIRS is suitable: (i) for a wide variety of populations (e.g., clinical patients, infants, elderly people), (ii) for bedside monitoring, and (iii) for those experimental situations that cannot be easily recreated within the physical constraints of an fMRI scanner because require the volunteer to have unconstraint physical movements (Scholkmann et al., 2014a, Quaresima and Ferrari, 2016). In fact, while motion artifacts can represent a major obstacle both for fMRI and electrophysiological techniques, such as electroencephalography (EEG) measurements, fNIRS is more robust against this issue and thus more suitable for tasks involving unconstrained physical movements. The development of wireless, miniaturized and fiberless fNIRS systems, opens up the way to more ecological applications in neuroscience, especially for those situations in which experiments conducted in the real-world are needed (Burgess et al., 2006, McKendrick et al., 2016, Pinti et al., 2015a).

For instance, mental workload and situation awareness in augmented reality wearable displays (ARWDs) are assessed traditionally by questionnaires administered during task probes, pauses or at the end of the experiment. However, these measures are less ecologically valid than measures taken in dynamic situations with mobile participants. Thanks to the new generation of wearable fNIRS devices, we have demonstrated their applicability for monitoring prefrontal cortex activation in freely moving subjects outside the lab (Pinti et al., 2015a); and recently, others (McKendrick et al., 2016) were able to assess mental workload and situation awareness during navigation in ARWDs in similar naturalistic situations. Wearable fNIRS devices can also be used in non-invasive Brain Computer Interface (BCI) systems to detect task-related brain activations in less restrained situations and control external devices for e.g. neuro-rehabilitation, communication or motor restoration (see Naseer and Hong (2015) for a review). In addition, monitoring brain activity in real life scenarios may also be particularly important in the study of executive functions, the high-level processes used to control and organise other mental processes in order to enable flexible goal-directed behaviour (Gilbert and Burgess, 2008, Lezak, 1995, Miller and Cohen, 2001). Previous studies have suggested that standard lab-based neuropsychological tests may be insensitive to executive function difficulties of patients with frontal lobe lesions, which can be revealed in more naturalistic real-world tasks (Burgess et al., 2006, Shallice and Burgess, 1991). Motivated by this, our team demonstrated the feasibility of investigating one aspect of executive function (prospective memory) using a fiberless and wearable fNIRS system (Pinti et al., 2015a). This allowed the measurement of prefrontal cortex hemodynamics of freely moving participants performing a prospective memory experiment outside the lab.

So far, fNIRS has been used mostly to monitor functional brain activity in response to computer-based cognitive tasks in conventional laboratory settings. Given the slow nature of the hemodynamic response, fNIRS and fMRI lab-based protocols are designed very similarly (Strangman et al., 2002). Lab-based experiments are usually structured as event- or block-related designs, in which task periods are spaced out by low-level baseline periods and stimuli are repeated multiple times in order to maximize the Signal-to-Noise ratio (SNR).

In the early stages of fNIRS research, brain activation was assessed typically by visual inspection or application of thresholds to the signals (Benaron et al., 2000, Murata et al., 2002, Tak and Ye, 2014). However, in order to get more rigorous and statistically meaningful interpretation of fNIRS data, the main approaches that have been adopted to infer changes in functional activity are averaging techniques, General Linear Models (GLM) and data-driven methods. The averaging approach consists in averaging signals across task and rest periods and in assessing brain activation by statistically testing (e.g., through *t*-tests or ANOVAs) the difference between task and rest average values. The advantage of these methods is that they do not have to make very accurate assumptions about the timing and/or the shape of the haemodynamic signal; however, the disadvantage is that they do not make use of the high temporal resolution of fNIRS (Tak and Ye, 2014). By contrast, the GLM method overcomes this issue and considers the entire fNIRS time course, providing more statistical power. The GLM is a well-established regression approach widely used for fMRI data analysis (Friston et al., 1994a), which has been extended for fNIRS applications, as both techniques recover the hemodynamic response. In the GLM analysis, fNIRS data are regressed using a linear combination of explanatory variables (i.e., regressors) plus an error term. Such task-related regressors are created by convolving boxcar functions, which reflect the experimental design, with a hemodynamic response function (HRF). The beginning and end of each function event is coded by the shape of the boxcar function, or, in the limiting case of an event with duration zero, a delta function. The design matrix is comprised by task-related regressors plus a constant term and models the expected hemodynamic response to the assigned cognitive task. However, whilst the GLM method presents different advantages, assumptions have to be made on the shape and timing of the HRF (Tak and Ye, 2014). Other data-driven approaches have been proposed as well for the analysis of task-evoked activity measured through fNIRS, such as Principal Component Analysis (PCA), Independent Component Analysis (ICA) and Task-Related Component Analysis (TRCA). These methods do not make any a-priori hypothesis of the HRF shape and rely on the assumption that fNIRS data are a mixture of task-related and task-unrelated components (Tanaka et al., 2013). Through these approaches, fNIRS data are decomposed into independent components assuming statistical independence between source signals. Task-related components are then identified, for example using a threshold of the mean inter-trial cross-correlation (Patel et al., 2011), maximizing the inter-trial covariance (Tanaka et al., 2013) or maximizing both inter-trial correlations and the covariance between HbO_2_ and HHb (Tanaka et al., 2014).

However, in order to create the boxcar function in the GLM approach, to compute task and rest mean values in the averaging method or to calculate the inter-trial correlations in data-driven methods, the timing of the event onsets must be known. In lab-based experiments such a timeline is established and controlled, the trial order is known a-priori and all the stimuli timings are triggered and recorded. However, this is not necessarily the case in real-world experiments conducted outside the lab, which are designed to be more ecological and to mirror real-life situations, without predetermined and controlled stimulus presentation. Whilst rules and some explicit targets can be used, the timing control can be very unpredictable. For example, in our previous study (Pinti et al., 2015a), participants were asked to perform a task in which they were required to remember delayed intentions (i.e. prospective memory) whilst walking freely in an outdoor environment. They were left free to accomplish the task without significant restraints, encountering different type of stimuli (e.g., obstacles, people, sounds, and so on) while they walked, looked around, crossed the streets and interacted with the environment. In addition, inter-subject variability needs to be taken into account as each participant is exposed to different stimuli and can use his/her own strategy to accomplish the task. Functional events in the real-world thus originate from the integration of complex and highly variable behaviours, which may be hard to identify from e.g., the behavioural analyses of video recordings. The identification of the event onsets from video examinations can be extremely difficult, time consuming and, sometimes, inaccurate as, for instance, it can be hard to predict if the real functional event in a freely moving participant occurs when they see the target stimulus or when they reach it.

In order to automatically disentangle these events and improve the identification of various behavioural actions through assessment of behavioural data (such as video recordings), in this study we propose a novel GLM-based method for the Automatic IDentification of functional Events (AIDE) that statistically detects functional events directly from fNIRS neuroimaging data. Rather than taking the standard approach of starting with a predetermined experimental design and investigating the effects of its events on haemodynamic activity, here we take the opposite approach of starting with neuroimaging data and seeking to identify the occurrence of experimental events on the basis of it. This algorithm is based on the GLM model and identifies functional events by evaluating the best fit between different models of functional activity, assembled considering all the possible combinations of onset time and duration of the events, and the experimental fNIRS data. AIDE represents the first step prior to the analysis of fNIRS data by means of the method described above (i.e., block-averaging, GLM, PCA/ICA), identifying the event onsets necessary for those approaches. In this study we describe the algorithm and as proof-of-principle we apply it to both synthetic data and experimental fNIRS data from a conventional computer-based/lab-based experiment, and also to data gathered during a real-world experiment outside the laboratory.

## Methods

The “AIDE algorithm formulation” section briefly outlines the mathematical structure of the proposed algorithm. Its application to synthetic, lab-based and real-world data are also presented in different sections.

### AIDE algorithm formulation

The AIDE algorithm aims to estimate functional events from fNIRS data on the basis of the GLM-based least square fit analysis. The GLM expresses the observed response (Y) as a linear combination of explanatory variables (X) plus an error term (ε):

(1) Y=Xβ+ε

Y is a N×M matrix (N=number of time points; M=number of fNIRS channels) representing the measured fNIRS signals; the design matrix X is a N×W matrix composed by W-regressors: regressors constitute the model of functional activation and are computed by convolving a boxcar function with the canonical hemodynamic response function (HRF); The W×M regression coefficients matrix, β, indicates the contribution of each regressor to the observed responses (Y); the N×M error matrix, ε, includes the independent and identically distributed errors, with 0 mean and $\sigma^{2}$ variance ($\varepsilon \sim N(0,\sigma^{2}$I)), representing the residual variance in the observed responses not explained by the model.

In this algorithm, the boxcar function s(t) is created considering all the possible combinations of the event time location/duration, translating the event onset $t_{i}$ while increasing its duration $d_{j}$ throughout the experiment. More precisely, its amplitude (Eq. (2)) is set to 1 during event periods, e.g. from the event time location $t_{i}$ (i=1,2,…N) to its duration $t_{i}+d_{j}(j=1,2,\ldots ,(N-t_{i}))$, and 0 otherwise:

(2) $$s\left(t\right)=\{\begin{array}{ccc} 1 & \;t_{i}:t_{i}+d_{j} & i=1:N;j=1,\;2,\ldots ,(N-t_{i}))\\ 0 & otherwise & \end{array}$$

β-values are estimated through the least square estimation, i.e., through the minimization of the sum of squared error values (S):

(3) $$S=\sum_{i=1}^{N}{\left(Y-\hat{Y}\right)}^{2}=\sum_{i=1}^{N}{\left(Y-X\hat{\beta }\right)}^{2}$$

where $\hat{Y}$ represents the fitted response and $\hat{\beta }$ the estimated β-values. The estimated parameters $\hat{\beta }$ and their variance are calculated as:

(4) $$\hat{\beta }={(X^{T}X)}^{-1}X^{T}Y$$

(5) $$\mathrm{var}\left(\hat{\beta }\right)=\sigma^{2}{(X^{T}X)}^{-1}$$

If X is full rank, β-estimates are normally distributed ($\hat{\beta }\sim N(\beta ,\sigma^{2}{(X^{T}X)}^{-1}$)). In order to statistically infer functional activity and to test hypotheses on linear combinations of effects of interest (i.e., β-estimates), T-statistics are computed by defining a contrast vector c so that $c^{T}\hat{\beta }\sim N(c^{T}\beta ,\sigma^{2}c^{T}{(X^{T}X)}^{-1}c$). More precisely, T-statistics are calculated as follows:

(6) $$T=\frac{c^{T}\hat{\beta }-c^{T}\beta }{\sqrt{{{\hat{\sigma }}^{2}c}^{T}{(X^{T}X)}^{-1}c}}$$

where the variance ${\hat{\sigma }}^{2}$ is:

(7) $${\hat{\sigma }}^{2}=\frac{{\left(Y-X\hat{\beta }\right)}^{T}(Y-X\hat{\beta })}{N-p}$$

P-values are then computed by comparing the T-values with the $T_{N-p}$ Student's T-distribution with N−p degrees of freedom, where p=rank(X) (Uga et al., 2014, Friston et al., 1994b, Monti, 2011).

AIDE identifies functional events under the assumption that β-estimates are indicators of the goodness of fit between the functional activation model (X) and the fNIRS experimental data (Y). All the possible combinations of event onsets and durations are thus tested and the one corresponding to the best fit (i.e., the best estimation of the regressors amplitude) is marked as functional event. More precisely, each combination onset/duration is individually inserted into the GLM procedure for the estimation of the corresponding β-value and t-value; then, at the end of all the possible combinations, all the estimated parameters from the GLMs are combined together and the functional events detection procedure is applied. GLM-based analyses of fNIRS data are influenced by serial autocorrelations present in fNIRS signals that can reduce the accuracy of the estimation of the hemodynamic response and can lead to inflated statistics. Serial correlations arise from the high sampling rates of fNIRS systems, in addition to systemic changes and motion artifacts that contaminate fNIRS signals (Barker et al., 2016). In order to minimize the impact of serial autocorrelations, we have adopted the following pre-processing steps prior the application of AIDE: (i) motion artifacts correction, (ii) fNIRS data denoising to remove high frequency physiological noise (e.g., heart rate, breathing rate) and to reduce systemic influences, and (iii) data down-sampling to 1 Hz using a spline interpolation. AIDE is then applied to the post-processed fNIRS data. The pre-processing steps adopted in this study are explained in detail in Sections 3.2–3.4. However, the pre-processing steps are independent from AIDE. Therefore, prior its application, one could use any pre-processing flow (e.g., filter parameters, down-sampling methods, motion artefact correction and systemic interference correction) that better suits the particular study. To further reduce the impact of systemic interferences (Tachtsidis and Scholkmann, 2016) and to avoid false positives and/or false negatives, prior to the GLM fitting procedure described above, the correlation-based signal improvement (CBSI, Cui et al., 2010) method is applied as a first step in the AIDE algorithm. The CBSI method reduces the systemic components in the fNIRS signals maximizing the anti-correlation between HbO_2_ and HHb (Haeussinger et al., 2014, Scholkmann et al., 2014b) as in typical functional brain activity (Obrig et al., 2000) and combining them into an ‘fNIRS activation signal’. The resulting ‘activation signal’ (Scholkmann et al., 2014a) is a weighted linear combination of HbO_2_ and HHb that allows a researcher to (i) operate on one signal simultaneously containing information on both HbO_2_ and HHb, (ii) denoise fNIRS signals from confounding factors and (iii) reduce the difficulties associated with different time delays between HbO_2_ and HHb responses (Kirilina et al., 2013). In this way, a more robust identification of functional events can be achieved.

**Fig. 1:**
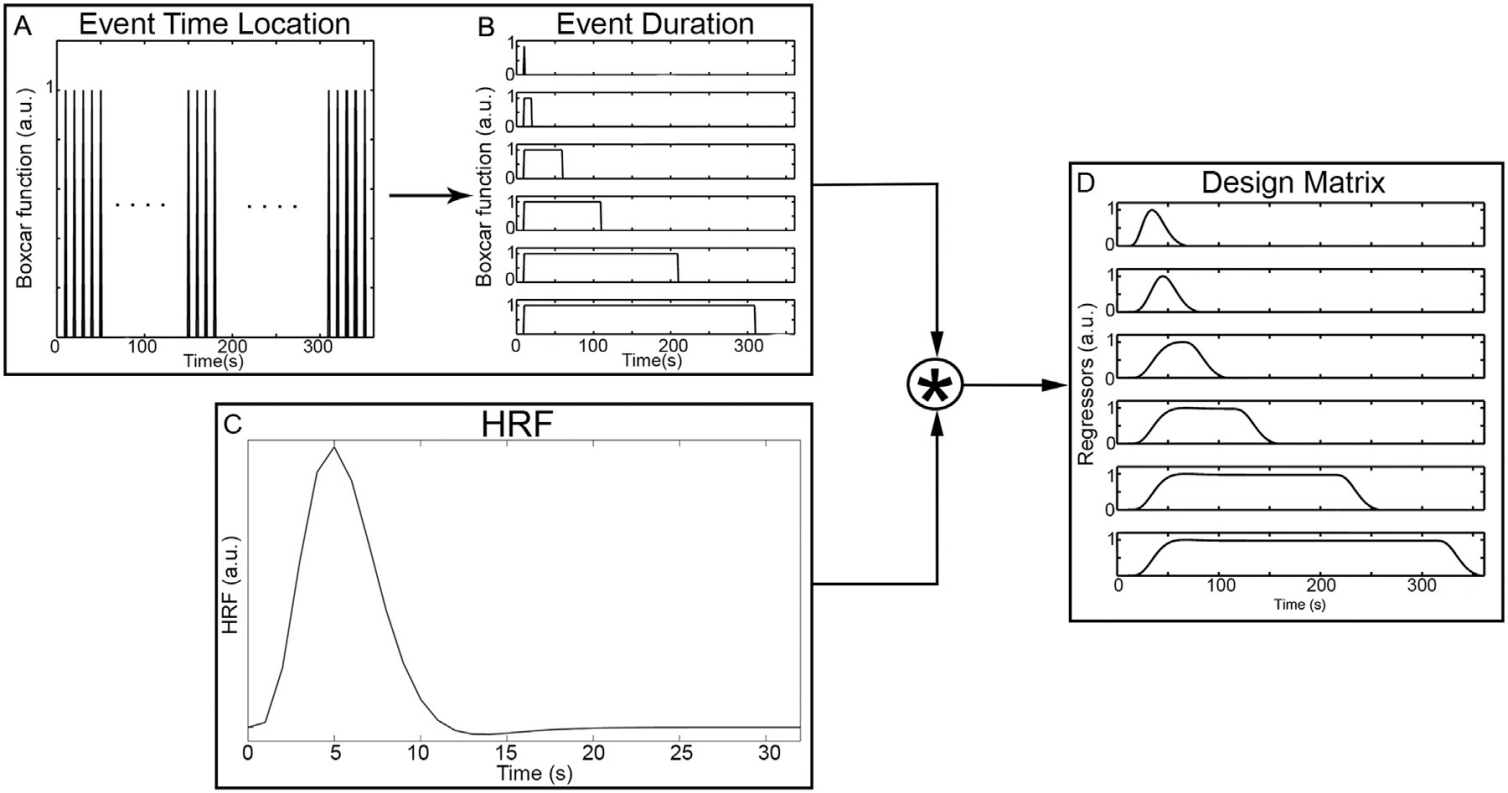
Boxcar function and design matrix computation. Boxcar functions are created by translating the event onset using 1-s steps throughout the experiment duration (A) while increasing the boxcar duration at 1-s steps (B). Each boxcar function is convolved with the HRF (C) to compute the design matrix (D).

In order to test all the possible combinations of time-location/duration of the events, boxcar functions (Eq. (2)) are created by translating each event onset with 1-second steps throughout the experiment (Fig. 1A) and, for each time location, its duration is increased with 1-s steps (Fig. 1B) either until the end of the experiment or until a maximum value that can be set by the user assuming that i.e. a hemodynamic response would not be sustained for more than 5 min.

**Fig. 2:**
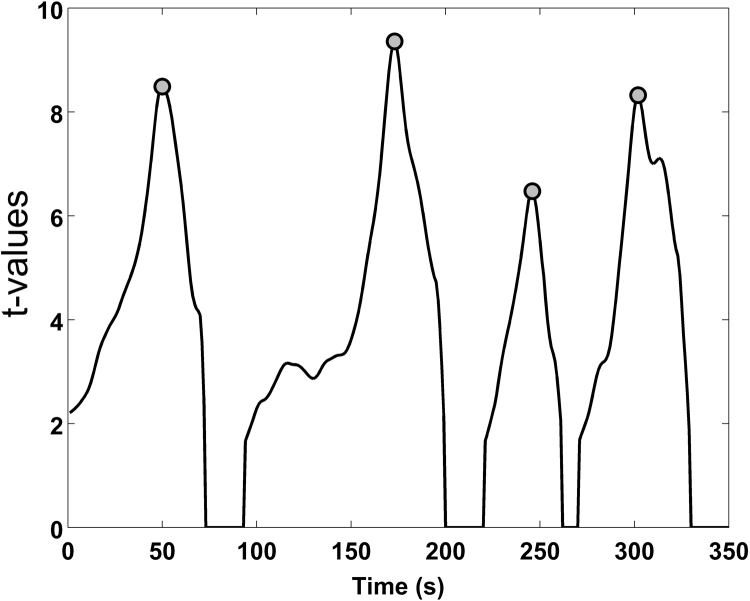
Events onset identification. An example of a t-value signal is represented by the black line while the peak-values representing functional events identified through the AIDE algorithm are shown as grey dots.

Each boxcar function, corresponding to a certain event onset and duration, is convolved with the canonical HRF (Fig. 1C). The chosen HRF is composed by a linear combination of two gamma functions (Handwerker et al., 2004, Uga et al., 2014, Friston et al., 1998), a positive one modelling the response, and a negative one the undershoot:

(8) $$h\left(\tau_{p},t\right)=\frac{t^{\tau_{p}}e^{-t}}{\left(\tau_{p}\right)!}-\frac{t^{\tau_{p}+\tau_{d}}e^{-t}}{A\left(\tau_{p}+\tau_{d}\right)!}$$

where $\tau_{p}$ indicates the first peak delay and $\tau_{d}$ the undershoot delay. These parameters were respectively set to 6 s and 10 s respect to the stimulus onset, as for typical fMRI/fNIRS studies, while the amplitude ratio between the first and the second peak (A) was set to 6 s (Uga et al., 2014). More precisely, one boxcar function at a time is entered into the GLM. This is equivalent to a correlation analysis as one regressor is tested each time. The design matrix (X) is composed by a constant term and the model used to fit the activation signal computed by convolving the particular boxcar function with the HRF (Fig. 1D); the corresponding β-values are estimated through the least square estimation (Eq. (4)) and the corresponding t-values are computed as well (Eq. (6)). After repeating this procedure for all the boxcars, the estimated β-values and t-values from all the GLMs are combined together to identify the functional events trough an iterative procedure. For each onset, non-significant t-values (p>0.05) are rejected and the maximum t-value and its corresponding duration (i.e., the best fit) are selected. This process ends with a time-course of the t-values (Fig. 2), where each time point identifies an event with its estimation of best time-location/duration. Additional examples of t-values time series resulting from this procedure showing the event detection procedure are included in Supplementary Section 2–4 of Supplementary material 1.

**Fig. 3:**
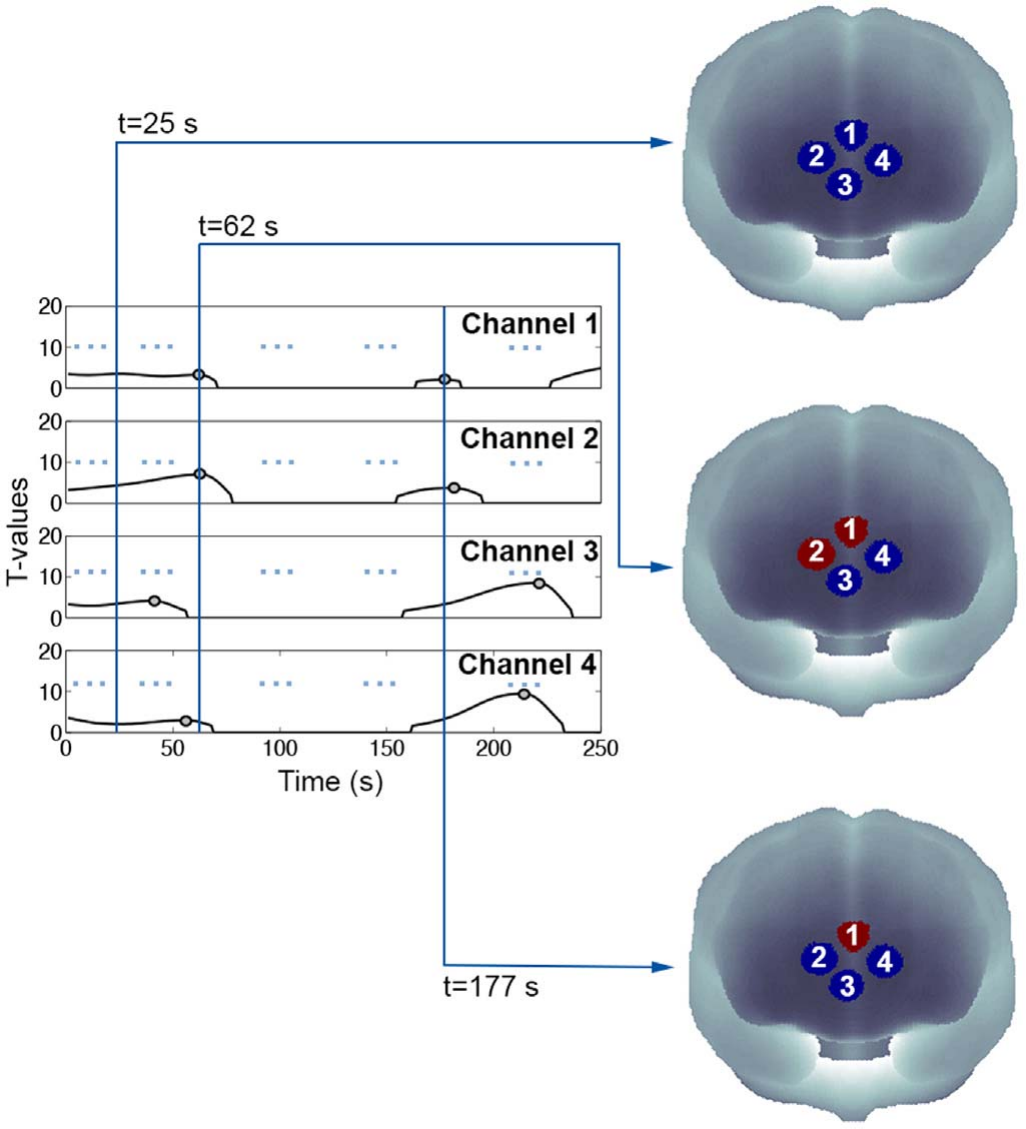
Example of binary maps development process using synthetic data. Binary maps are created for each signal time point and show the activated channels (red circles) and non-active channels (blue channels) as predicted by AIDE. t-values signals are represented by black lines while functional events are identified by grey dots.

Peak-values are extracted from on the t-value signal (Fig. 2, grey dots) and they correspond to the best combination of onset time-location/duration, i.e., the best fit between the activation signal and the activation model. This is done by finding local maxima, i.e. all the points that are at least a certain amount (equal to the 50% of the t-values signal range) above the surrounding points, using the Matlab function ‘*peakfinder’* (Yoder, 2016). T-values peaks associated with bigger boxcars containing smaller boxcars are excluded and only the smaller ones are considered. In fact, when multiple functional events occur in close proximity to each other, the overall hemodynamic response results from the summation of single-event HRFs. Time points are classified as functional events when the remaining t-values exceed a threshold with a certain significance level (p_thresh_), which has been established on the basis of numerical simulations (see Section 3.1). In case of multichannel fNIRS data, AIDE is applied for each channel individually, with one regressor per channel corresponding to each tested boxcar. Once all the boxcars are tested and functional events are detected for all the channels, a binary brain map for each time point of the experimental condition is produced in order to visualize the channels involved in each functional event (Fig. 3).

In order to account for the multiplicity of tests performed by AIDE, our results were corrected using the False Discovery Rate (FDR) approach (Benjamini–Hochberg procedure (Benjamini and Hochberg, 1995)) with a maximum false discovery rate equal to the p_thresh_ determined through the simulations (see Section 3.1). In fact, in case of multiple statistical tests, it is important to limit false discovery rate and to control error rates; for this reason, FDR is highly suitable as demonstrated by others (Cai and Liu, 2016, Hu et al., 2010).

### AIDE application to fNIRS synthetic data

In order to test performance of the developed algorithm and to determine the optimal p_thresh_, numerical simulations (N=1000) with synthetic data were performed. AIDE was applied to time courses simulating a block design, an event-related design and a mixed-design experiments. The mixed-design experiment includes both event- and block-type of activity and it is a better representation of real-world experiments. For all the simulated experiments, HbO_2_ and HHb synthetic time courses were sampled at 1 Hz and the duration was set at 600 s (Tanaka et al., 2013). HbO_2_ synthetic signals were generated including a task-related component, a noise component and a physiological task-independent component emulating the Mayer wave. The latter was not included in the HHb data generation as previous studies demonstrated that, compared to HbO_2_, HHb is less affected by systemic confounds (Tachtsidis and Scholkmann, 2016, Kirilina et al., 2012) such as Mayer waves and cardiac pulsations (Tachtsidis et al., 2004, Haeussinger et al., 2014). In fact, veins walls are composed of less smooth muscle than arteries (Fink, 2009), being less innervated by sympathetic fibers and thus less influenced by autonomic regulations (Tachtsidis and Scholkmann, 2016, Haeussinger et al., 2014). HHb synthetic signals were accordingly composed of a task-related component and a noise component.

**Fig. 4:**
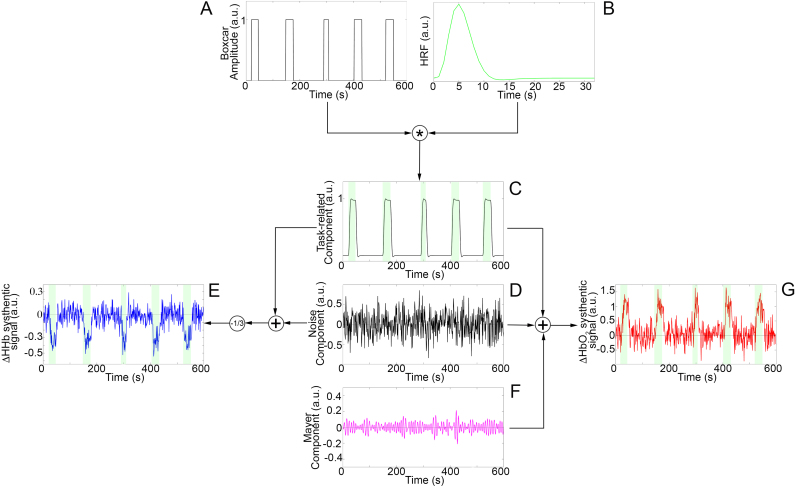
Block-design synthetic data generation. The task-related component (C) is created through the convolution of the boxcar function (A) with the HRF (B). HbO_2_ synthetic signals (G) are composed by the task-related component (C), a noise component (D) and a physiological task-independent component (F). HHb synthetic signals (E) include the task-related component (C) and a noise component (D). Green areas mark the task periods.

For the block-design experiment (Fig. 4), the task-related component (Fig. 4C) was modelled as a boxcar function (Fig. 4A, Eq. (2)) convolved with the double-gamma HRF (Fig. 4B, Eq. (8)). The boxcar amplitude was set to 1 (a.u.) during task periods and to 0 during rest periods. The boxcar function was designed to include five task blocks (Tanaka et al., 2013) separated by rest periods. Rest and task block durations were randomly sampled from the Uniform Distribution U(20,120) s and U(15,30) s, respectively.

**Fig. 5:**
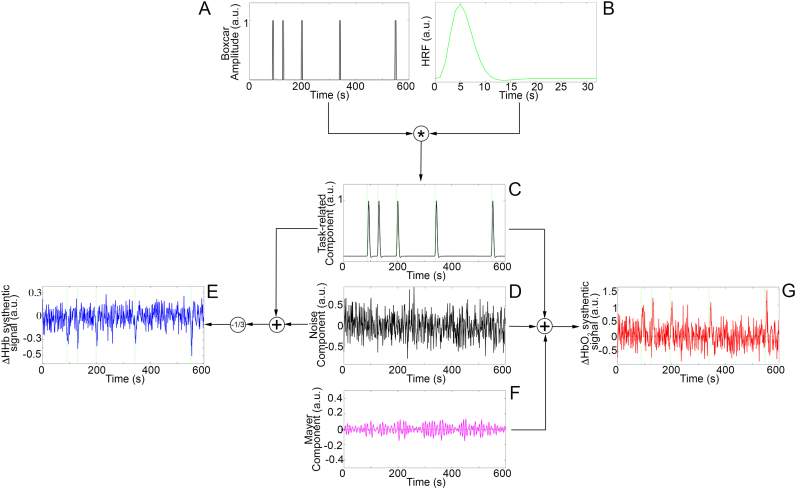
Event-related design synthetic data generation. The task-related component (C) is created through the convolution of the boxcar function (A) with the HRF (B). HbO_2_ synthetic signals (G) are composed of the task-related component (C), a noise component (D) and a physiological task-independent component (F). HHb synthetic signals (E) include the task-related component (C) and a noise component (D). Green areas mark the task periods.

For the event-related design experiment (Fig. 5), the task-related component (Fig. 5C) was modelled as a three seconds and a unitary amplitude boxcar function (Fig. 5A, Eq. (2)) (Tanaka et al., 2013) convolved with the double-gamma HRF (Fig. 5B, Eq. (8)). It included five randomly sampled task events separated by rest periods with a minimum duration of 5 s.

The mixed-design experiment included both blocks and events as described above; the task-related component was modelled as a unitary amplitude boxcar function. In this case, the number of functional events were randomized in order to test AIDE performance with a different number of onsets. More precisely, the number of blocks and events were both randomly sampled from the uniform distribution U(1,4), with a minimum of 2 events to a maximum of 8 in total for each synthetic time series. Blocks duration was randomly sampled from the uniform distribution U(15,30) s while events duration was set at 3 s.

**Fig. 6:**
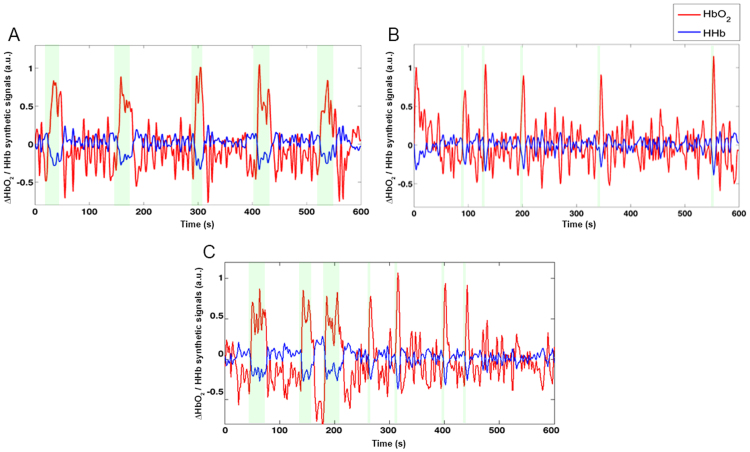
Examples of synthetic signals. Pre-processed synthetic HbO_2_ (red) and HHB (blue) signals corresponding to the simulated block (A), event-related (B) and mixed-design (C) experiments. Green areas mark the task periods.

For all the three simulated experiments, the noise component consisted of a randomly generated Gaussian noise with 0 mean and variance 0.3^2^ (Figs. 4D and 5D). The HHb synthetic signal was generated by adding together the task-related and the noise components and its amplitude was reduced by a -1/3 factor (Figs. 4E and 5E) considering that HHb changes have smaller amplitude than HbO_2_ (Gagnon et al., 2012) and the two chromophores are anti-correlated (Obrig et al., 2000). A physiological task-independent component emulating the Mayer wave was further included in the HbO_2_ data generation (Figs. 4F and 5F). The Mayer wave component was modelled as a band-pass filtered (Butterworth, 4th order, [0.08–0.15] Hz (Kirilina et al., 2013)) random Gaussian noise with 0 mean and 0.15^2^ variance, as Mayer wave oscillations are usually centred at 0.1 Hz with a broad frequency peak (Tachtsidis et al., 2004). The HbO_2_ synthetic signal was thus generated by adding together the task-related, the noise and the Mayer wave components (Figs. 4G and 5G). Synthetic data from the three simulated experiments were pre-processed (Fig. 6) through a 3rd order Butterworth band-pass filter with cut-off frequencies of 0.008–0.2 Hz.

The filtered synthetic HbO_2_ and HHb signals were combined into the activation signal using the CBSI method after which we applied AIDE. A total of 1000 synthetic data have been simulated for each experimental design. For each possible boxcar, AIDE tested the null hypothesis $H_{0}:c^{T}\hat{\beta }=0$, where c=[01], N=600, W=2, M=1. Algorithm performance was tested using different p_thresh_ (0.05, 0.01, 0.001, 0.0001, 0.00001, 0.000001). The events identified by AIDE were classified as “correct events” if they fell within a 6 s window centred around the a-priori event onset. No limits were set to the duration of the identified event. The optimal p_thresh_ was determined through a ROC analysis (Fawcett, 2006), on the base of the specificity and sensitivity of the algorithm across all the simulations and experimental designs as the best compromise between False Positive Rate and True Positive Rate. Mean accuracy was computed across all the simulations using the optimal p_thresh_, for the block, event-related and mixed-design experiments. Mean event onset and duration difference between the identified functional event onsets and the real event onsets was evaluated, as well.

Additional simulations were carried out to test the performance of AIDE with mixed-design simulated experiments with different levels of noise and boxcar amplitudes. We varied the ratio of the noise standard deviation to the boxcar amplitude to assess AIDE sensitivity in case of different activity levels and noise conditions. These results are described in detailed in Supplementary Section 1 of the Supplementary Material 1.

### AIDE application to lab-based fNIRS data

The feasibility of AIDE in the identification of functional events was then investigated by applying the developed method to real experimental fNIRS data previously published in Pinti et al. (2015b), using the p_thresh_ determined through the simulations (see Section 3.1). Here we present the algorithm application to data from a single participant (P1, healthy female, 25 years old), as it aims to identify functional events at the single-subject level and not to infer functional activity in response to a cognitive task at a group-level. As a further test of the performance of AIDE in real experimental fNIRS data, we applied AIDE to an additional participant (healthy female, 20 years old) who underwent the same lab-based experiment as P1. We present these results in Supplementary Section 4 of the Supplementary Material 1.

**Fig. 7:**
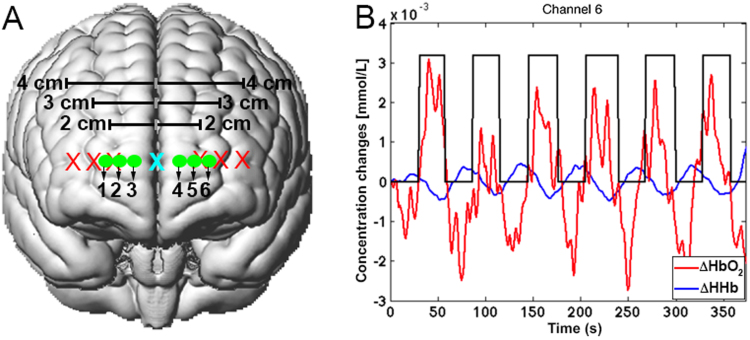
Channels configuration. Optodes (red crosses=sources; cyan cross=detector) and channels locations over the prefrontal cortex (A). (B) Example of pre-processed HbO_2_ (red line) and HHb (blue line) signals corresponding to channel 6. The black boxcar indicates the experimental protocol.

fNIRS data were collected during a typical block-designed mathematical task (see Pinti et al. 2015b for further details). Briefly, the experiment consisted of six task blocks where participants were asked to perform three consecutive subtractions (e.g., 17,235–271 = 16,964, 16,964–271 = 16,693, 16,693–271=16,422) per block. Task periods were spaced out by 30 s rest periods. Prefrontal cortex activity was monitored through a frequency-domain near-infrared spectroscopy system (Imagent, ISS Inc., Champaign, IL). Laser sources were time-multiplexed and sampling frequency was set to 10 Hz. Optodes (6 laser sources and one detector) were symmetrically arranged in a multi-distance configuration, as shown in Fig. 7A, with source-detector distances of 2, 3 and 4 cm, creating six measurement channels.

To minimize the effect of serial autocorrelation on GLM analyses, unpreprocessed HbO_2_ and HHb data were corrected for motion artifacts using a wavelet-based correction algorithm (Molavi and Dumont, 2012) and physiological noise was reduced using a 3rd order Butterworth band-pass filter with cut-off frequencies of 0.008–0.2 Hz. HbO_2_ and HHb pre-processed data were down-sampled to 1 Hz using a spline interpolation and then combined using the CBSI method to one signal called ‘fNIRS activation signal’ (Scholkmann et al., 2014a). To compute the accuracy of the developed method on lab-based data, we first used the GLM analysis implemented in the NIRS-SPM software package (Ye et al., 2009) to establish if statistically significant increase in the fNIRS activation signal occurs for each task block individually. We thus modelled each task block as an individual regressor and contrasted each task regressor versus the previous rest phase regressor. Results for this analysis with six contrasts for the six individual regressors were corrected for multiple comparisons by means of the Bonferroni correction. We then applied AIDE on the same activation signals from the six channels. For all the boxcar combinations, AIDE tested the null hypothesis $H_{0}:c^{T}\hat{\beta }=0$, where c=[01], N=374, W=2, M=6. AIDE results were corrected for multiple comparisons using the FDR approach with a maximum false discovery rate of 0.0001 (p_thresh_). In order to compare the performance of the NIRS-SPM GLM analysis with a-priori experimental onsets against the AIDE-identified onsets, the fNIRS activation signals from the six channels were entered into the NIRS-SPM software package (Ye et al., 2009). GLM analysis was used to assess activation by contrasting the task blocks regressor versus the rest periods regressor using both the onsets identified by AIDE and the a-priori experimental defined onsets.

### AIDE application to real-world fNIRS data

In order to assess the feasibility of the AIDE algorithm in the detection of functional events in real-world neuroimaging data, the method was applied to a single case study (P2, healthy male, 20 years old) from a real-world fNIRS data recorded during a naturalistic prospective memory (PM) experiment, using the p_thresh_ determined through the simulations.

The PM experiment was conducted outside the lab in a typical London location (Queen Square, London WC1N, U.K.). The experimental protocol is described in detail in Pinti et al. (2015a). Briefly, the ecological prospective memory paradigm was designed according to the principles of a typical prospective memory experiment in cognitive neuroscience (for review see Burgess et al., 2009, Burgess et al., 2011). The term “prospective memory” refers to the abilities required to create, maintain, and executive intended actions after a delay period during which the participant is fully occupied with some other activity. Everyday life examples might be remembering to pass on a message to a colleague when you next see them, or remembering to make a telephone call at the end of a meeting. In a typical neuroimaging experiment of this kind, there are at least three key conditions (e.g., Burgess et al., 2001, Burgess et al., 2003). First, there is a baseline condition which exposes the participant to the stimuli that they are going to experience during the other two conditions, but where there is little other cognitive demand. Second, there is an “ongoing task” condition. This consists of the activity in which the participant is engaged during the delay period between creating an intention and when they encounter a prospective memory cue (which should signal intention execution). Third, there is the prospective memory condition, which consists of the participant performing the ongoing task exactly as in the ongoing condition, but where they have the additional demand of responding in a novel way to specified prospective memory cues, which do not directly interrupt ongoing task performance, but have instead to be noticed by the participant. The logic of the experiment is that the contrast between the baseline and ongoing conditions reveals neural activity related to the ongoing task; and the block-level contrast between ongoing activity and the prospective memory conditions (or event-related contrast between ongoing activity performance imbedded in the prospective memory blocks and event-related activity when prospective memory cues are detected and responded to) demonstrates activity related to various aspects of delayed intention maintenance and execution. In the prospective memory paradigm used here (the “Queen Square PM task”), there were a series of four different ongoing tasks which involved detecting and counting particular objects in the environment (e.g. the number of push-button door entry systems) which were counterbalanced for order across participants and prospective memory conditions. There were also two prospective memory conditions, where the participants were required to respond in a particular way to cues in the environment. In the social PM (sPM) condition, they were required to notice and respond to one of the experimenters, who placed themselves in various positions in the environment. The response was a “fist-bump” greeting. In the non-social PM (nsPM) condition, they were required to respond to parking meters in the same way. There were five social cues and approximately the same number of non-social cues. The order of the prospective memory conditions was counter balanced across participants. The overall aim of the experiment was to determine whether fNIRS could detect different patterns of neural activity within prefrontal cortex according to whether the intention was related to social or non-social prospective memory cues. Participants engaged in cognitively demanding tasks while they walked around outside, in an area of central London, UK, known as “Queen Square”. In some conditions, they were also required to “fist-bump” either parking meters or a person. In addition, there were several baseline tasks (not described here) at the start and the end of the experiment. There was also a repeated presentation of the ongoing-task only block which always occurred after the prospective memory conditions. This is a procedure typically used to measure the residual cognitive processing that occurs after intention have been executed and no longer need to be responded to. It is known as the “contaminated ongoing condition (OGc)”, and is contrasted with the ongoing condition that is presented before any prospective memory instruction, which is known as the “uncontaminated ongoing condition (OG)”. The entire experimental session was filmed by three video cameras for post-hoc examination of participants’ behaviour.

**Fig. 8:**
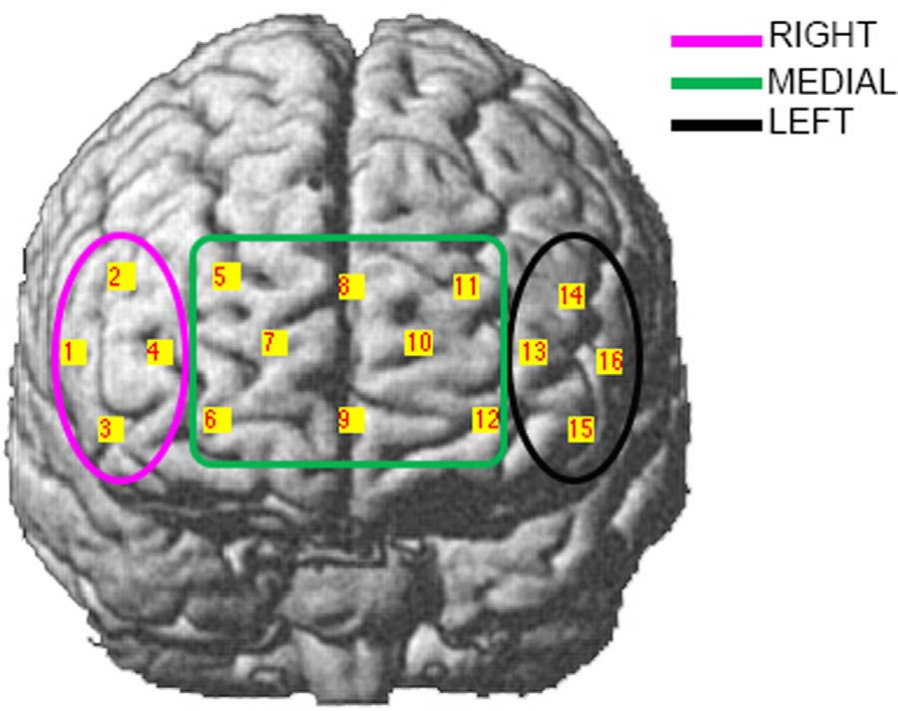
Channels configuration and regions of interest. Regions of interest were defined on the basis of the Brodmann Area atlas.

Prefrontal cortex activity was continuously monitored by means of a wireless and fiberless 16-channels fNIRS system (WOT, Hitachi High-technologies Corporation, Japan) (Atsumori et al., 2009). The WOT headset was covered by a black shading cap to improve light shielding and minimising the exposure of the optical detectors to ambient light from the environment. Optodes and channels locations were digitized using a 3D magnetic digitizer (Fastrak, Polhemus). The real coordinates of the digitized locations were converted into the MNI coordinates and registered onto a brain template in the MNI space (Fig. 8) (Okamoto and Dan, 2005, Singh et al., 2005, Ye et al., 2009). Channels were grouped by defining regions of interest (ROIs) with the help of the Brodmann Area (BA) atlas (see Supplementary Material 2). More precisely, channels belonging to right BA 45–46 (Channels 1, 2, 3, 4) were labelled as RIGHT (Fig. 8, magenta); channels belonging to left BA 45–46 (Channels 13, 14, 15, 16) were labelled as LEFT (Fig. 8, black); channels belonging to BA 10–11 (Channels 5, 6, 7, 8, 9, 10, 11, 12) were labelled as MEDIAL (Fig. 8, green).

A linear detrend between the first and the last baseline was applied to the unpreprocessed real-world HbO_2_ and HHb signals. To account for serial autocorrelations, HbO_2_ and HHb signals were corrected for motion artifacts using a wavelet-based correction algorithm (Molavi and Dumont, 2012), band-pass filtered (0.008–0.2 Hz) and then down-sampled to 1 Hz using a spline interpolation. Corrupted channels (channels not measuring due to stray light interference) were excluded from AIDE results. Considering that brain activity was monitored on freely moving participants and that fNIRS signals are influenced by systemic changes (Tachtsidis and Scholkmann, 2016), heart rate and breathing rate changes were measured through a monitoring belt (Bioharness, Zephyr Technology Ltd., USA). The relative contribution of each physiological signal to each fNIRS channels was assessed through a GLM using the physiological data as regressors. The weight parameters (β) were thus estimated through the least square method (Eq. (4)). Systemic changes interference was then reduced by subtracting the heart and breathing rate signals multiplied by the corresponding β value for each channel from the fNIRS signals. (Sato et al., 2016). HbO_2_ and HHb signals were then combined by means of the CBSI method to one fNIRS activation signal and analysed with AIDE. For each iteration, AIDE tested the null hypothesis $H_{0}:c^{T}\hat{\beta }=0$, where c=[01], W=2, M=14 and N=357 for the OG condition, N=473 for the sPM condition, N=413 for the nsPM condition and N=373 for the OGc condition. Accuracy was investigated through the behavioural analysis of video recordings by corresponding the timings of the algorithm-recovered functional events with participants’ behaviour.

## Results

The “Synthetic fNIRS Data” section presents the results obtained from the application of the AIDE algorithm to synthetic fNIRS data, while the “Lab-based fNIRS Data” and “Real-world fNIRS Data” sections show the results of the use of the AIDE algorithm for the identification of functional events in a conventional block-design experiment and a real-world PM task.

### Synthetic fNIRS data

ROC parameters resulting from the ROC analysis on the block, event-related and mixed-design simulated experiments are summarized in Table 1.

**Table 1:** ROC parameters for the simulated block, event-related and mixed-design experiments.

	**Block Design**		**Event-Related Design**		**Mixed Design**	
p*thresh*	**Sensitivity**	**Specificity**	**Sensitivity**	**Specificity**	**Sensitivity**	**Specificity**
**0.05**	88.68%	99.69%	96.74%	99.11%	92.23%	99.44%
**0.01**	88.68%	99.82%	96.74%	99.57%	92.19%	99.75%
**0.001**	88.68%	99.87%	96.74%	99.84%	91.82%	99.88%
**0.0001**	88.66%	99.88%	96.64%	99.92%	90.89%	99.91%
**0.00001**	88.66%	99.89%	96.38%	99.95%	87.92%	99.92%
**0.000001**	88.64%	99.90%	95.30%	99.96%	83.67%	99.93%

AIDE performed better than a random classifier (Area under the curve (AUC)=50%) achieving a AUC=94.28% for the block experiment, AUC=98.34% for the event-related experiment and AUC=96.06% for the mixed-design experiment. In all cases, p=0.0001 resulted the optimal p_thresh_ as a compromise between False Positive Rate and True Positive Rate in both experimental designs. For p_thresh_=0.0001, performance results are summarized in Table 2.

**Table 2:** Results of the performance of the AIDE algorithm in the simulated block- and event-related design experiments.

*p*_*thresh*_*=0.0001*	**Block Design** (Mean ± s.d.)	**Event-related Design** (Mean ± s.d.)	**Mixed Design** (Mean ± s.d.)
**Accuracy**	89±14%	97±8%	91±13%
**Onset Difference**	1.00±0.01 s	0.62±0.02 s	0.72±0.03 s
**Duration Difference**	1.23±0.02 s	0.24±0.03 s	0.81±0.01 s

The mean accuracy (mean ± s.d., number of identified events/number of real events) across the 1000 simulations was of 89±14% for the block design experiment, 97±8% for the event-related design experiment and 91±13% for the mixed-design experiment. The mean difference (mean ± s.d.) between the real event onsets and the identified event onsets resulted of 1.00±0.01 s for the block experiment, 0.62±0.02 s for the event-related experiment and 0.72±0.03 s. In terms of the corresponding identified duration, the mean duration difference (mean ± s.d.) between the real event and the identified event resulted of 1.23±0.02 s for the block experiment, 0.24±0.03 s for the event-related experiment and 0.81±0.01 s for the mixed-design experiment. Examples of t-values time series and functional events computed by AIDE on synthetic data simulating the block, the event-related, and the mixed-design experiments, the corresponding identified boxcars and models are included in Supplementary Section 2 of Supplementary Material 1. Results referring to the additional simulations we have performed investigating AIDE sensitivity with different noise and activation levels are reported in Supplementary Section 1 of Supplementary Material 1. Briefly, these additional simulations demonstrated that AIDE remains a good classifier with good specificity also in case of higher noise levels and smaller activation amplitudes. However, the increase in noise levels is reflected as some loss in sensitivity (see Supplementary Table 1 and 2 of Supplementary Material 1).

Concerning the computational duration, AIDE took 133 s to run for each synthetic signal (length=600 s; fs=1 Hz) using a laptop running Windows 10 64 bit, with a 7th generation processor (Intel Core i7-7500U), 8GB of RAM and 1TB hard drive.

### Lab-based fNIRS data

Results referring to P1 are summarized in the following tables. AIDE was applied individually to the fNIRS activation signals from all six channels and, in order to investigate its performance and compute its accuracy, we determined for each task block whether brain activity occurs or not. We thus entered the fNIRS activation signals from the six channels in the GLM analysis in the NIRS-SPM software package and we contrasted each task block vs. the previous rest phase. All the six task blocks showed significant activation (Table 3, p<0.008 Bonferroni corrected for multiple comparisons).

**Table 3:** Results of the NIRS-SPM GLM analysis for the assessment of significant functional activity at single task-block level and t-values for each channel and each contrast.

	**Ch. 1**	**Ch. 2**	**Ch. 3**	**Ch. 4**	**Ch. 5**	**Ch. 6**
**Contrasts**	**t(58)**	**t(58)**	**t(58)**	**t(58)**	**t(58)**	**t(58)**
**Task#1 vs Rest#1**	4.99	4.80	3.41	4.45	5.21	5.26
**Task#2 vs Rest#2**	3.29	3.51	3.03	3.39	4.57	4.19
**Task#3 vs Rest#3**	5.38	5.60	5.24	4.44	5.77	5.65
**Task#4 vs Rest#4**	4.52	4.94	5.14	3.74	6.01	5.11
**Task#5 vs Rest#5**	3.85	4.70	3.86	3.56	4.87	3.89
**Task#6 vs Rest#6**	2.89	4.29	3.67	3.39	5.86	4.20

All the contrasts computed on the fNIRS activation signals achieved statistical significance (p<0.008 Bonferroni corrected for multiple comparisons).

Table 4 shows the onset of each task block established in the experimental design and the corresponding onset identified by the AIDE algorithm using the p_thresh_=0.0001.

**Table 4:** Results of the performance of the AIDE algorithm in the lab-based block design experiment.

**Real Task Onset (s)**	**Identified Task Onset (s)**					
**(s)**	**Ch 1**	**Ch 2**	**Ch 3**	**Ch 4**	**Ch 5**	**Ch 6**
32	31	34	35	33	33	32
88	*	*	*	*	*	*
147	152	149	148	147	148	148
207	208	207	207	207	208	208
270	271	271	271	271	271	270
330	*	324	326	324	329	326

The asterisks mark the identified onsets excluded from further analyses as they did not achieve significance for the assigned p_thresh_=0.0001. Results are FDR corrected for multiple comparisons.

Table 5 presents the duration of each task block established in the experimental design and the corresponding duration identified by the AIDE algorithm using the p_thresh_=0.0001 established through the simulations (see Section 3.1).

**Table 5:** Duration of the task blocks identified by AIDE.

**Real Task Duration (s)**	**Identified Task Duration (s)**					
**(s)**	**Ch 1**	**Ch 2**	**Ch 3**	**Ch 4**	**Ch 5**	**Ch 6**
**26**	24	22	21	22	22	23
**28**	*	*	*	*	*	*
**30**	24	27	28	19	21	27
**32**	21	22	23	22	24	25
**30**	16	16	17	16	18	19
**28**	*	28	25	23	24	27

The asterisk marks the identified onset excluded from further analyses as it did not result in significance for the assigned p_thresh_=0.0001. Results are FDR corrected for multiple comparisons.

Mean onset and duration differences between the experimental design and the AIDE-identified events are reported in Table 6.

**Table 6:** Mean ± Standard deviation of differences between the a-priori experimental onsets and durations and AIDE-identified onsets and durations.

	**Ch 1**	**Ch 2**	**Ch 3**	**Ch 4**	**Ch 5**	**Ch 6**
**Mean Onset Difference (s)**	2.00±2.00	2.20±2.28	1.80±1.64	1.60±2.51	1.00	1.20±1.64
**Mean Duration Difference (s)**	8.25±5.32	6.20±5.67	6.40±4.56	8.80±4.21	7.40±3.44	5.00±4.00

AIDE identified 5/6 task onsets, resulting in an accuracy (number of identified onsets/number of real onsets) of 83,3% for all the channels except for Channel 1 for which 4/6 events were identified (Accuracy=66,7%). We then compared the performance of the NIRS-SPM GLM analysis using the a-priori onsets established in the experimental design and the AIDE-identified onsets, contrasting the task blocks regressor versus the rest periods regressor. The task block periods not identified by AIDE were considered as rest. Results are summarized in Table 7.

**Table 7:** Comparison between the GLM analysis performed using the a-priori and the AIDE-identified onsets.

	**Ch. 1**	**Ch. 2**	**Ch. 3**	**Ch. 4**	**Ch. 5**	**Ch. 6**
**Onsets**	**t(58)**	**t (58)**	**t (58)**	**t(58)**	**t(58)**	**t(58)**
**a-priori onsets**	9.95	10.51	10.49	8.61	12.40	11.56
**AIDE onsets**	7.96	11.22	10.25	10.74	10.89	11.38

t-values are reported for each channel.

The onsets identified through AIDE generally improve the GLM analysis reflected by the increase in the t-values as it improves the fit between fNIRS signals and the model. For Channel 1, the GLM analysis with AIDE onsets performs slightly worse. In fact, for Channel 1, AIDE identified 4/6 blocks as functional events with the remaining two assigned as rest periods. The decreased t-values are related to the longer rest periods that are characterized by – not significant but still present – increases in the activation signal that reduce the significance of the contrast task versus rest. Examples of t-values signals computed by AIDE to recover functional events for participant P1 for the lab-based fNIRS data with the corresponding identified boxcars and models are in Supplementary Section 2 of the Supplementary Material 1. Results referring to an additional participant are included in Supplementary Section 3 of the Supplementary Material 1. Briefly, for this additional participant AIDE reached an average accuracy of 91,7%; in addition, AIDE recovered other events that did not reach significance for the single-task NIRS-SPM analysis and significantly improved the GLM analysis.

Concerning the computational duration, AIDE took 154 s to run using a laptop running Windows 10 64 bit, with a 7th generation processor (Intel Core i7-7500U), 8GB of RAM and 1TB hard drive.

### Real-world fNIRS data

**Fig. 9:**
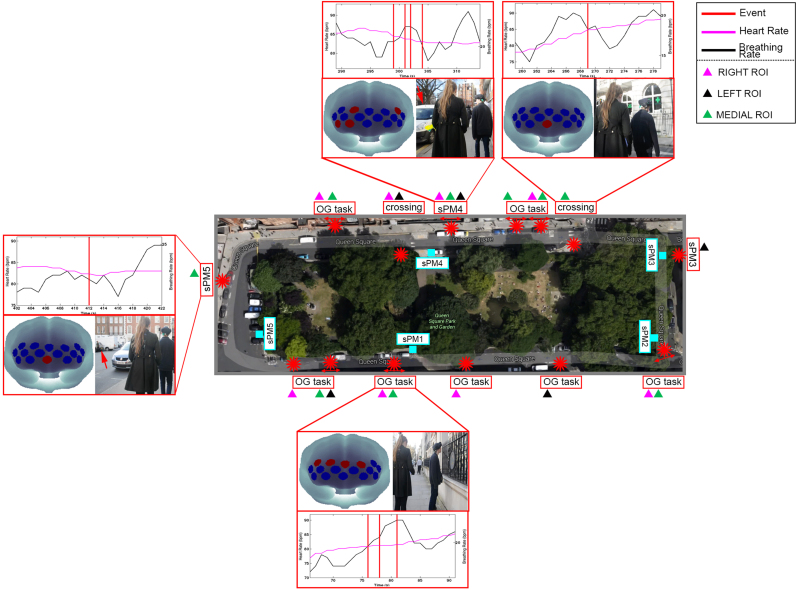
Results of the application of the AIDE algorithm to P2 sPM condition. Positions of the sPM targets within the square are represented by light blue squares. The detected functional events are identified by red asterisks. Functional events are corresponded with participant's behaviour and the involved ROIs are reported as well as brain maps showing the specific responding channels (red circles) and the non-involved channels (blue circles). Functional events are marked with red lines on the heart and breathing rate signals.

The results presented in Fig. 9 refer to participant P2 during the social prospective memory condition (sPM). Results corresponding to the ongoing-only condition (OG), non-social prospective memory condition (nsPM) and the contaminated ongoing condition (OGc) are reported in Supplementary Section 5 of the Supplementary Material 1.

In Fig. 9 the Queen Square map (i.e., the experimental area of the real-world PM task) is provided and the location within the square of the identified events are represented by red asterisks. Binary maps showing the channels involved in each identified event, together with the heart rate and breathing rate time series are plotted. For participant P2, Channels 4 and 16 were excluded because they were highly corrupted. In Fig. 9 we also illustrate the brain maps of the channels identified to be active (on - red circles) or not (off - blue circles) for each recovered event, together with a video capture showing what the participant was doing at that time point. Example of t-values signals computed by AIDE to identify functional events, the recovered boxcars and corresponding models for all the four experimental conditions are included in Supplementary Section 5 of the Supplementary Material 1.

**Fig. 10:**
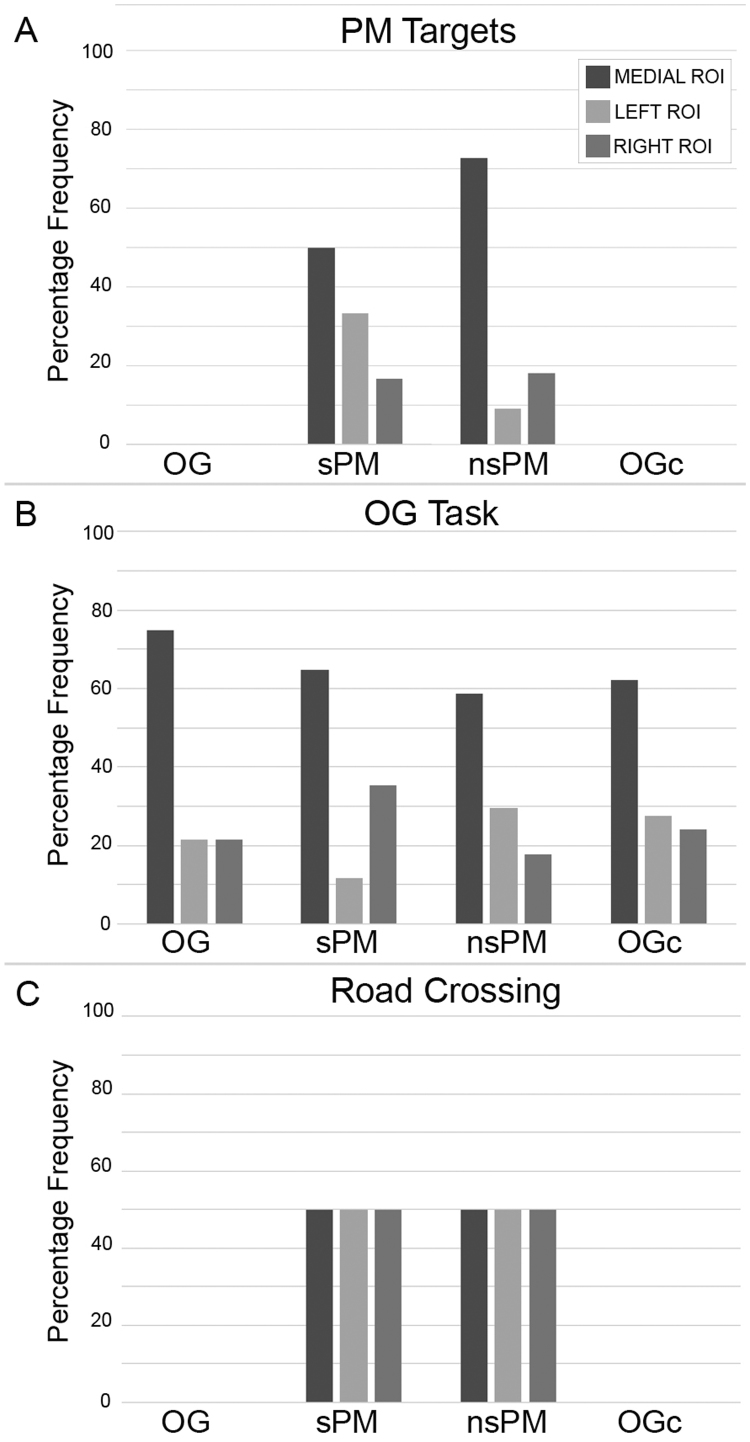
ROI analysis results. MEDIAL, LEFT and RIGHT ROIs percentage involvement in the PM Targets processing, OG Task and Road Crossing across the four experimental conditions are shown in panel A, B and C, respectively.

The participant's performance and actions were evaluated through the analysis of video recordings. More precisely, P2 responded to 4/5 sPM targets, missing the first sPM cue (sPM1), and to 6/6 nsPM targets. Video recordings were also used to match the identified functional events with participant's behaviour. AIDE detected all the social PM hits (3/4, Accuracy=75%) and 3/6 nsPM hits (Accuracy=50%, see Supplementary Figure 13 of Supplementary Material 1). Results suggest that functional events to the PM tasks are more likely to occur when the participant first notices the PM targets, both social and non-social, rather than when he reaches them. In addition, AIDE algorithm identified other activity-based prospective memory actions, such as the road crossing and other moments when the participant was performing the OG task such as counting the number of items around the square. Brain regions responding to the different tasks were investigated, as well. In particular, channels were grouped into RIGHT, LEFT and MEDIAL ROIs (Fig. 8). ROI results are summarized in Fig. 10.

For the processing of PM targets (Fig. 10A), we found that the MEDIAL ROI was mostly involved in the reaching of sPM and nsPM cues. For the execution of the OG task (Fig. 10B) across the four experimental conditions, we found that the MEDIAL ROI was the main region that consistently was involved while the RIGHT and LEFT ROIs were implicated to a lesser extent. The three ROIs were equally responding during the identified road crossing events (Fig. 10C).

AIDE required 355 s to run for the OG condition (16 channels; signal length=357 s; fs=1 Hz), 355 s to run for the sPM condition (16 channels; signal length=473 s; fs=1 Hz), 478 s for the nsPM condition (16 channels; signal length=413 s; fs=1 Hz) and 355 s to run for the OGc condition (16 channels; signal length=357 s; fs=1 Hz). AIDE was executed using a laptop running Windows 10 64 bit, with a 7th generation processor (Intel Core i7-7500U), 8GB of RAM and 1TB hard drive.

## Discussion

In this paper, we describe a methodology that aims to identify functional events directly from fNIRS neuroimaging data, and we present a proof-of-principle of operation with synthetic, lab-based and real-world fNIRS data. Even though this method represents - to the best of our knowledge - the first attempt to recover brain functional events directly from fNIRS data, preliminary results suggest that “brain-first” rather than “behaviour-first” analysis is in principle possible and can provide a powerful tool to improve the analysis of real-world neuroimaging experiments. This new approach can help to solve some issues with real-world neuroimaging and, in particular, the automatic recovery of functional events in more ecological and unstructured experiments, where the onset timeline is not established a-priori and is difficult to identify from participants’ behaviour. In fact, while conventional neuroimaging experiments are usually computer-based and structured as controlled block or event-related designs, real-world protocols are designed in a more ecological manner, with as few experimental constraints as possible, in order to mimic everyday life demands and settings as much as possible. Predicting and identifying stimuli onsets in the real-world can thus be extremely difficult as the context is uncontrolled and stimulus presentation is not pre-established. For instance, in case of experiments conducted outside the lab on freely moving participants (Pinti et al., 2015a), subjects are left free to walk and to accomplish the task, dealing with a wide variety of stimuli (e.g., obstacles, road crossing, people, sounds, etc.) and using different strategies. Participants’ behaviour is usually assessed through the analysis of video recordings of the experimental session. However, for such experiments this procedure can be time-consuming with a high risk of inaccurate events identification. The novel AIDE algorithm was developed with the aim of providing a tool to support the behavioural analysis of video recordings by statistically detecting functional event onsets from fNIRS data. This is achieved using a GLM approach (Friston et al., 1994a) to evaluate the similarity of fNIRS signals with a model of hemodynamic response. More precisely, the AIDE algorithm works on a ‘fNIRS activation signal’ created as a first step through the combination of HbO_2_ and HHb data by means of the CBSI method (Cui et al., 2010). This step helps to produce one signal (i.e., the ‘fNIRS activation signal’) containing information on both HbO_2_ and HHb. We are currently working on approaches beyond the CBSI that combine fNIRS data into a single signal that will allow better statistical identification of brain activity both at individual and group level analysis. In this particular example, we measured and used physiological data as regressor to denoise fNIRS signals (Sato et al., 2016) prior to CBSI to account for the systemic effects on the data. This is an important step for resolving false positives as recently discussed by Tachtsidis and Scholkmann (2016) but also to minimize the effect of serial autocorrelations in fNIRS signals (Barker et al., 2016). However, as AIDE is applied on post-processed data and thus independent from pre-processing steps, different pre-processing techniques to reduce serial autocorrelations, motion errors and physiological noises more suitable for other contexts can be applied.

### Synthetic fNIRS data

In this study, AIDE was first applied to synthetic fNIRS data in order to optimize the algorithm parameters and to test its accuracy. More precisely, this was done by generating synthetic fNIRS signals corresponding to simulated block, event-related and mixed-design experiments, as real-world protocols are usually uncontrolled and can involve both block and event-type of stimuli. For the simulated block and event-related design experiments, we used a fixed number of functional events (5 for each synthetic signal) and a constant boxcar amplitude; the noise and the Mayer components as well as the onset and the duration (for the block-design experiment) of the stimuli were randomized across all the synthetic signals, providing evidence of AIDE performance in different circumstances. To further prove AIDE robustness, we varied the number of functional events, noise levels and boxcar amplitudes for the mixed-design simulated experiment. Different significance levels (p_thresh_) were tested in order to optimize the algorithm detection performance. A ROC analysis was thus employed to determine the optimal p_thresh_ (0.0001) corresponding to the best compromise between False Positive Rate and True Positive Rate for block, event-related and mixed-designs. Results (Table 1) showed that the AIDE algorithm performs better than a random classifier, achieving an AUC>>0.5. This was also the case of synthetic data with higher noise levels and smaller boxcar amplitudes (see Supplementary Section 1 of Supplementary Material 1), for which AIDE lost in sensitivity but not in specificity and classification performance. In addition, the optimal p_thresh_ among the tested thresholds and between the three simulated experiments corresponded to 0.0001, which ensures a good balance between False Positives and False Negatives. For p_thresh_=0.0001, AIDE achieved an accuracy of 89% for the block-design experiment, 97% for the event-related experiment and 91% for the mixed-design experiment (Table 2).

### Lab-based fNIRS data

Once the p_thresh_ was established, the feasibility of the AIDE algorithm for recovering functional events in lab-based neuroimaging data was tested by applying the algorithm to fNIRS signals recorded during a computer-based mathematical task (Pinti et al., 2015b). In order to evaluate the performance of the AIDE algorithm in lab-based fNIRS data, brain activity at single task block level was first assessed through a conventional GLM-based analysis implemented into the NIRS-SPM toolbox (Ye et al., 2009). This was done to identify the number of task blocks in which brain activity occurred. A statistically significant increment in the activation signal was observed for all the six task repetitions (Table 3) for participant P1. AIDE identified 5/6 functional events (Table 4) corresponding to an accuracy of 83,3% for all the channels except for Channel 1 for which AIDE achieved an accuracy of 66.7%. Similar results were obtained for the additional participant we tested (see Supplementary Section 4 of Supplementary Material 1), where AIDE reached an average accuracy of 91,7%. However, in both cases, the difference in the onset localization through AIDE compared to the a-priori experimental onsets is higher than the one we found for the synthetic data (see Section 3.1). This might be related to the fact that in the synthetic data generation hemodynamic responses are “artificially” added into the signal, with a pre-determined onset; on the contrary, in real data, brain activity onset can be delayed/anticipated, thus not always reflecting the a-priori experimental design specifications. This was reflected in the improvement of the GLM-based analysis using the AIDE-identified onsets compared to the a-priori experimental onsets (Table 7 and Supplementary Table 7 of Supplementary Material 1). AIDE can therefore help in the identification of effective brain activity without any a-priori hypothesis by taking the opposite approach, that is starting from neuroimaging data to identify the effects of the cognitive task on hemodynamic activity.

### Real-world fNIRS data

For the evaluation of the AIDE algorithm in real-world experiments, the AIDE functional events were corresponded with participant's behaviour analysed through the video recordings of the experimental session. Fig. 9 shows the results of the AIDE algorithm on a representative participant (P2) for the sPM condition (see Supplementary Section 5 for the OG, nsPM and OGc conditions). For the PM conditions, the main finding is that the AIDE algorithm was able to identify all 3/4 functional events (Accuracy=75%) corresponding to the 4 sPM targets (Fig. 9, P2 missed the sPM1) and 3/6 functional events (Accuracy=50%) for the nsPM cues (Supplementary Figure 13 of the Supplementary Material 1). Second, this event did not always occur when the participant reached the sPM or the nsPM target, but when he actually spotted the target and approached it, in agreement with our previous results (Pinti et al., 2015a) showing anticipatory hemodynamic responses. Third, other events related to the execution of the OG task (i.e., counting the number of doorbells around the square) as well as activity-based PM actions were detected, too (see Supplementary Section 5). Although the presented results are preliminary and refer to one participant, they are very promising since highly localized brain activity is observed. This is particularly important because systemic physiological regulation processes can confound fNIRS signals (Tachtsidis and Scholkmann, 2016), resulting in a global widespread and non-specific haemodynamic response across the whole measurement area (Zhang et al., 2016). More precisely, the analysis of the ROIs revealed major involvement of the MEDIAL ROI (BA 10–11) in the execution of the OG task across the four experimental conditions (Fig. 10B). This result is in agreement with previous computer-based neuroimaging PM studies highlighting the stronger involvement of medial BA 10 during the performance of OG tasks compared to PM tasks (Burgess et al., 2011). Consistently, left and right PFCs were recruited during the execution of PM tasks such as reaching social and non-social PM targets (Fig. 10A).

### Limitations and future directions

To the best of our knowledge, the method presented here represents the first form of analysis developed specifically for real-world neuroimaging. One possible area of application can be the identification of functional events in the fNIRS-based brain-computer interface (BCI) field, and in those situations involving no artificial task designs, such as in the monitoring of neurological patients undergoing neuropsychological tests or during neurorehabilitation or in the study of social interactions (Scholkmann et al., 2013) in ecological contexts. It would also be interesting to evaluate AIDE sensitivity in a wider range of unstructured and task-free experiments, with less disguisable and subtler brain activity responses. However, AIDE can be used also for detecting functional events in unstructured fMRI experiments. In fact, it recovers the hemodynamic responses from neuroimaging data and, as both fNIRS and fMRI are based on neurovascular coupling, it can be relevant for both techniques.

One limitation of this work is the lack of more detailed and immediate information of participants’ behaviour for a more accurate validation of the presented method within the PM experiment. For example, eye tracking systems and GPS data could provide more accurate descriptions of participants’ behaviour and gaze and the path covered within the square. (Note: a GPS device was used in the PM study; however, we were unable to receive GPS signals in that particular experimental area). Further studies will be performed incorporating eye tracking and GPS data and further improvements will be implemented in order to augment the specificity of event detection and to automatically classify the identified events with the help of pattern recognition and machine learning analyses. For instance, the possibility to include additional regressors (e.g., nuisance regressors) in the tested AIDE models to improve the event detection and to account for other variable will be evaluated. Moreover, the implementation of additional and advanced methods to reduce serial autocorrelations (e.g. pre-whitening, precoloring (Barker et al. 2016; Ye et al., 2009)) within AIDE will be investigated. The possibility of recovering functional events in real-time will be evaluated as well as the possibility to make the AIDE algorithm available as a Matlab toolbox or integrated in existing fNIRS software packages.

## Conclusion

In this work, we presented a proof-of-principle method that recovers functional events from fNIRS neuroimaging data. The novel AIDE algorithm was developed with the aim of providing a tool that statistically detects functional event onsets from fNIRS data in case of unstructured task designs such as experiments conducted in the real-world where the events timeline is unknown in advance. We tested the AIDE performance on synthetic fNIRS signal and on fNIRS data collected during a typical lab-based block-design experiment and an ecological real-world prospective memory task, achieving a high accuracy in all the three cases.

Real-life situations represent the new frontier for the study of cognitive functions in more ecologically valid settings. With the availability now of wearable and wireless fNIRS devices, in this study we aimed to fill the gap between real-life testing and functional neuroimaging, providing new solutions for the analysis of functional neuroimaging data recorded in real-life scenarios.

## Conflict of interest

The authors have no conflict of interest.
