# Supplementary material for: A novel GLM-based method for the Automatic IDentification of functional Events (AIDE) in fNIRS data recorded in naturalistic environments
Source: Neuroimage. 2017 Jul 15;155:291–304. doi: 10.1016/j.neuroimage.2017.05.001 (PMC5518772; doi:10.1016/j.neuroimage.2017.05.001)
Supplement: Supplementary file 1 — Supplementary material [file mmc1.docx]

**1. Real-world fNIRS Data channels location**

For the real-world prospective memory experiment described in section 2.4 of the main text, prefrontal cortex hemodynamics was continuously measured by means of a wireless and fiberless 16-channels fNIRS device. Channels locations were digitized using a 3D magnetic digitizer (Fastrak, Polhemus) and converted and overlapped onto a brain template in the MNI space (Supplementary Figure 1). Anatomical regions covered by channels were determined with the help of the Brodmann Area (BA) atlas, giving the MNI coordinates of the channels as input to the atlas using the function implemented in the NIRS-SPM software package. The anatomical structure overlaid by the channels and the corresponding BA are reported in Supplementary Table 1.

**
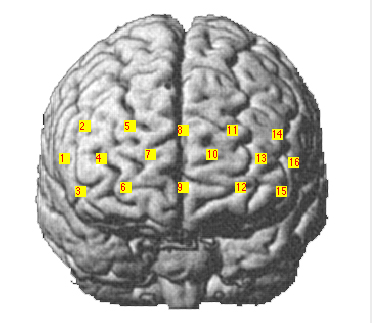
**

**Supplementary Figure 1. Channels location onto the MNI brain template**

| **Channels** | **Brodmann Area** | |
| --- | --- | --- |
|  | **Anatomical Structure** | **Area** |
| Channel 1 | Pars triangularis Broca's area | 45 |
| Channel 2 | Pars triangularis Broca's area | 45 |
| Channel 3 | Dorsolateral prefrontal cortex | 46 |
| Channel 4 | Dorsolateral prefrontal cortex | 46 |
| Channel 5 | Frontopolar area | 10 |
| Channel 6 | Orbitofrontal area | 11 |
| Channel 7 | Frontopolar area | 10 |
| Channel 8 | Frontopolar area | 10 |
| Channel 9 | Frontopolar area | 10 |
| Channel 10 | Frontopolar area | 10 |
| Channel 11 | Frontopolar area | 10 |
| Channel 12 | Orbitofrontal area | 11 |
| Channel 13 | Dorsolateral prefrontal cortex | 46 |
| Channel 14 | Pars triangularis Broca's area | 45 |
| Channel 15 | Dorsolateral prefrontal cortex | 46 |
| Channel 16 | Pars triangularis Broca's area | 45 |

**Supplementary Table 1. Channels anatomical structure and label according to the Brodmann Area atlas.**
