# Supplementary material for: A novel GLM-based method for the Automatic IDentification of functional Events (AIDE) in fNIRS data recorded in naturalistic environments
Source: Neuroimage. 2017 Jul 15;155:291–304. doi: 10.1016/j.neuroimage.2017.05.001 (PMC5518772; doi:10.1016/j.neuroimage.2017.05.001)
Supplement: Supplementary file 2 — Supplementary material [file mmc2.docx]

**1. AIDE application to additional synthetic data simulating a mixed-design experiment**

Additional numerical simulations were performed with the aim of testing AIDE in different conditions of noise and activity. In the main text, AIDE performance was tested on synthetic data simulating a block-design experiment, an event-related design experiment and a mixed-design (both events and blocks) experiment, with fixed boxcar amplitudes. The number of events for the block and event-related simulated experiments were constant and set at 5, while it was randomized for the mixed-design, sampling the number of both blocks and events from the uniform distribution U(1,4) (see Section 2.2 in the main text). The noise component was modelled as random Gaussian noise with 0 mean and standard deviation 0.3. The Mayer component was included only in the HbO_2_ data generation and was created by band-pass filtering (Butterworth, 4^th^ order, [0.08-0.15] Hz) a random Gaussian noise with 0 mean and 0.15^2^ variance. Data were sampled at 1 Hz and the signal length was set at 600 s.

Here we present additional synthetic data simulating mixed-design experiments where we varied the noise component variance e the boxcar amplitude in order to test the performance of AIDE with different noise and activity conditions. A ROC analysis was performed and AIDE sensitivity, specificity and the AUC of the ROCs were evaluated for all the new simulations at different p_thresh_ (0.05, 0.01, 0.001, 0.0001, 0.00001, 0.000001).

**1.1 Variable noise levels**

In order to examine the performance of AIDE with different noise levels while fixing the boxcar amplitude, we generated two sets of 500 synthetic signals each (Simulation 1 and Simulation 2) simulating a mixed-design experiment described in Section 2.2 of the main text, using the same boxcar amplitudes and the Mayer components. The standard deviation σ of the noise was set to 0.5 in Simulation 1 and 0.7 in Simulation 2, being the 50% and the 70% of the boxcar amplitude (A=1 a.u., held constant) respectively. Examples of synthetic time series are shown in Supplementary Figure 1.

**
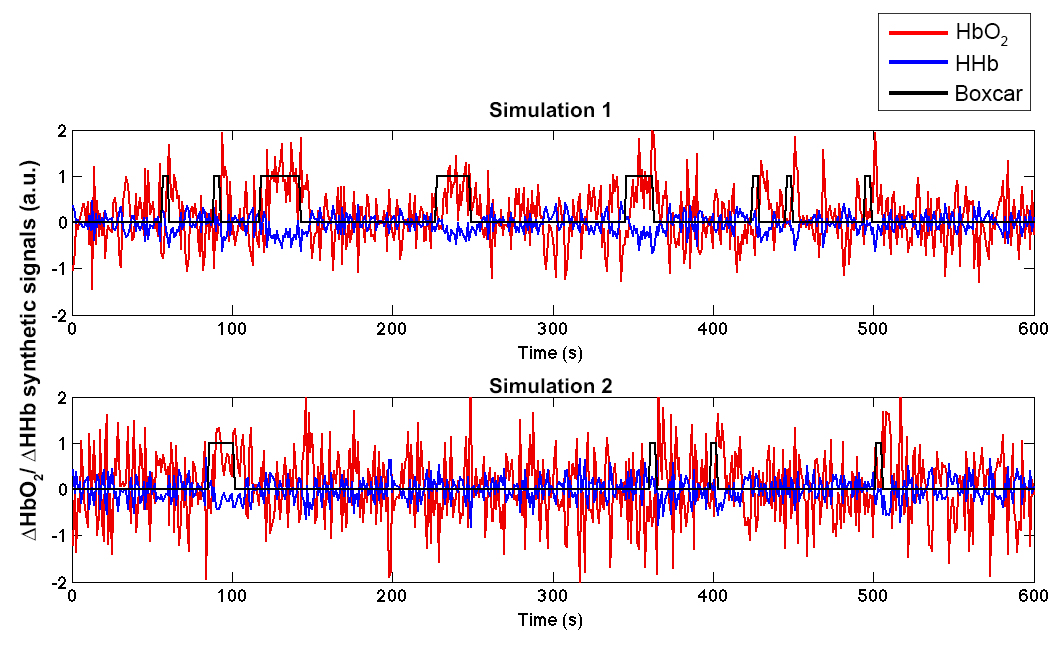
**

**Supplementary Figure 1. Example of raw synthetic fNIRS signals generated in Simulation 1 (first row) and Simulation 2 (second row).**

AIDE performance was evaluated at different p_thresh_ for both Simulation 1 and Simulation 2 and results of the ROC analyses are presented in Supplementary Table 1.

|  | Simulation 1  σ=0.5; A=1  AUC=87.62% | | Simulation 2  σ=0.7; A=1  AUC=78.05% | |
| --- | --- | --- | --- | --- |
| p*thresh* | **Sensitivity** | **Specificity** | **Sensitivity** | **Specificity** |
| 0,05 | 75,71% | 98,91% | 57,10% | 98,52% |
| 0,01 | 75,28% | 99,33% | 54,87% | 99,01% |
| 0,001 | 72,91% | 99,64% | 49,80% | 99,41% |
| 0,0001 | 67,62% | 99,76% | 42,70% | 99,60% |
| 0,00001 | 61,36% | 99,80% | 36,12% | 99,71% |
| 0,000001 | 53,86% | 99,83% | 28,62% | 99,77% |

**Supplementary Table 1. Simulation 1 and 2: Sensitivity and specificity of AIDE at different p_thresh_.**

Using p_thresh_=0.0001 as a compromise between False Positive Rate and True Positive Rate, the mean duration difference between the real onset and the AIDE identified was 0.71±0.06 s for Simulation 1 and 0.89±0.12 s for Simulation 2. The corresponding onset difference resulted to be 0.65±0.03 s for Simulation 1 and 0.58±0.04 s for Simulation 2. The increase in noise levels impacts on AIDE sensitivity, yet still being highly specific to event detection. Also, the differences in the identified onsets and corresponding duration are comparable to the results obtained with lower noise levels (see Table 2 in the main text) and are smaller than 1 s even with higher noise variance.

**1.2 Variable noise levels and boxcar amplitudes**

In order to further evaluate the performance of AIDE in case of functional activations with smaller amplitudes, we run additional simulations simulating a mixed-design experiment using the same Mayer component as for Simulation 1 and 2, and varying the ratio of the noise level to the boxcar amplitude:

- Simulation 3: boxcar amplitude A=0.3 a.u.; noise standard deviation σ=0.1, being the 30% of the boxcar amplitude as for the mixed-design experiment described in Section 2.2 of the main text;
- Simulation 4: boxcar amplitude A=0.5 a.u.; noise standard deviation σ=0.25, being the 50% of the boxcar amplitude as for Simulation 2;
- Simulation 5: boxcar amplitude A=0.7 a.u.; noise standard deviation σ=0.49, being the 70% of the boxcar amplitude as for Simulation 3.

We generated 500 synthetic signals for each additional simulation. Examples of synthetic time series are shown in Supplementary Figure 2.

**
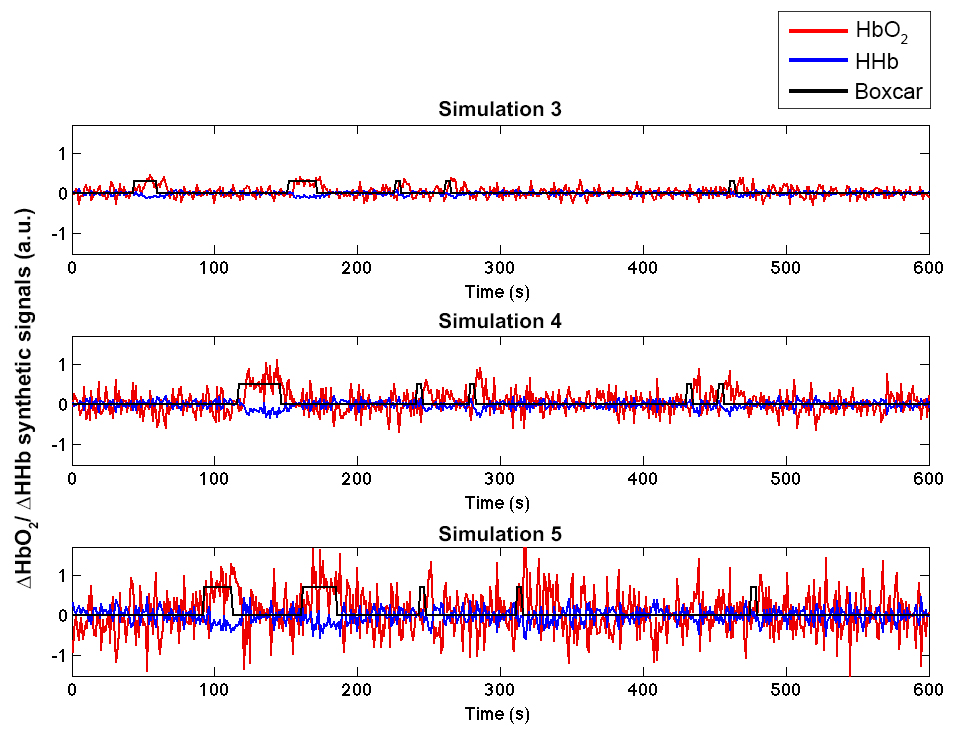
**

**Supplementary Figure 2. Example of raw synthetic fNIRS signals generated in Simulation 3 (first row), Simulation 4 (second row) and Simulation 5 (third row).**

AIDE performance was evaluated at different p_thresh_ for Simulation 3, Simulation 4 and Simulation 5. Results of the ROC analyses are summarized in Supplementary Table 2.

|  | Simulation 3  σ =0.1; A=0.3  AUC=94.18% | | Simulation 4  σ =0.25; A=0.5  AUC=86.18% | | Simulation 5  σ =0.49; A=0.7  AUC=78.03% | |
| --- | --- | --- | --- | --- | --- | --- |
| p*thresh* | **Sensitivity** | **Specificity** | **Sensitivity** | **Specificity** | **Sensitivity** | **Specificity** |
| 0,05 | 88,54% | 99,29% | 72,87% | 98,92% | 56,99% | 98,55% |
| 0,01 | 88,43% | 99,65% | 72,32% | 99,34% | 55,18% | 99,04% |
| 0,001 | 88,09% | 99,83% | 69,59% | 99,62% | 51,50% | 99,43% |
| 0,0001 | 86,01% | 99,88% | 65,02% | 99,73% | 45,16% | 99,62% |
| 0,00001 | 82,17% | 99,90% | 58,42% | 99,78% | 37,13% | 99,72% |
| 0,000001 | 76,90% | 99,90% | 51,41% | 99,82% | 29,97% | 99,78% |

**Supplementary Table 2. Simulation 3, 4 and 5: Sensitivity and specificity of AIDE at different p_thresh_.**

Mean duration difference between the real onset and the AIDE identified and the corresponding onset difference at the optimal p_thresh_=0.0001 were:

- Simulation 3: duration difference= 0.69±0.04 s; onset difference= 0.78±0.02 s;
- Simulation 4: duration difference= 0.70±0.07 s; onset difference= 0.62±0.03 s;
- Simulation 5: duration difference= 0.80±0.11 s; onset difference= 0.49±0.04 s.

This new set of simulations demonstrated that AIDE is able to detect functional events also in case of subtler brain activity, being highly specific to onset detection and with small differences in terms of onset identification and corresponding duration (<1 s).

**1.3 Conclusions on additional simulations**

Taken together, the results obtained from simulated data suggest that AIDE performance is related to the ratio of the noise levels to the boxcar amplitude rather than only to noise levels. We run six different simulations (mixed-design simulated experiment described in Section 2.2 of the main text and Simulation 1, 2, 3, 4 and 5) changing both noise levels and boxcar amplitudes while keeping their ratio constant between simulations pairs: mixed-design simulation (see Section 2.2 of the main text) and Simulation 3 (ratio=30%), Simulation 1 and 4 (ratio=50%), Simulation 2 and 5 (ratio=70%). The results for each pair of simulations are very similar in term of AUC, sensitivity, specificity and onset/duration differences, also when the synthetic activity has a lower amplitude and is thus less distinguishable in the signal. Although the sensitivity is reduced when the ratio is higher, AIDE remains highly specific, and a good classifier for all ratios, with AUCs>78%.

**2. AIDE results: t-values time series, identified boxcars and model**

In order to test performance of AIDE in different type of experimental designs, numerical simulations with synthetic data were performed. More precisely, AIDE was applied to the CBSI-derived fNIRS activation signals simulating a block-design experiment, an event-related design experiment and a mixed-design experiment (see Section 2.2 of the main text).

In this section, we present an example for each simulated experimental design described in Section 2.2 of the main text, illustrating the t-value signal used by AIDE to identify functional events, the recovered boxcar and corresponding model (Supplementary Figure 3, 4 and 5).


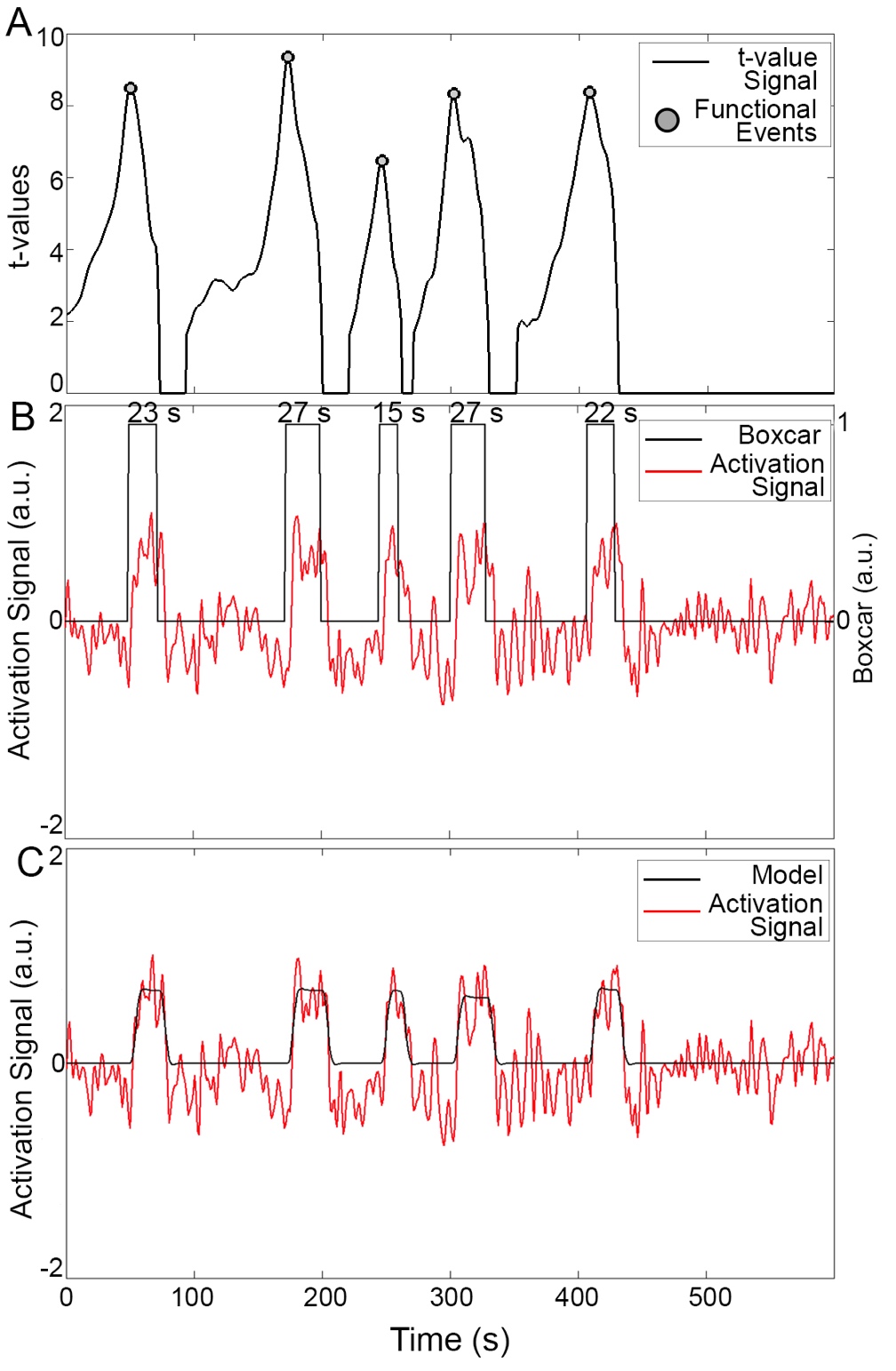


**Supplementary Figure 3. Example of t-values signal corresponding to a block-design synthetic signal with AIDE-identified functional events (A), the corresponding recovered boxcar and durations (B) and model (C) overlapped onto the synthetic CBSI-derived fNIRS activation signal.**

**
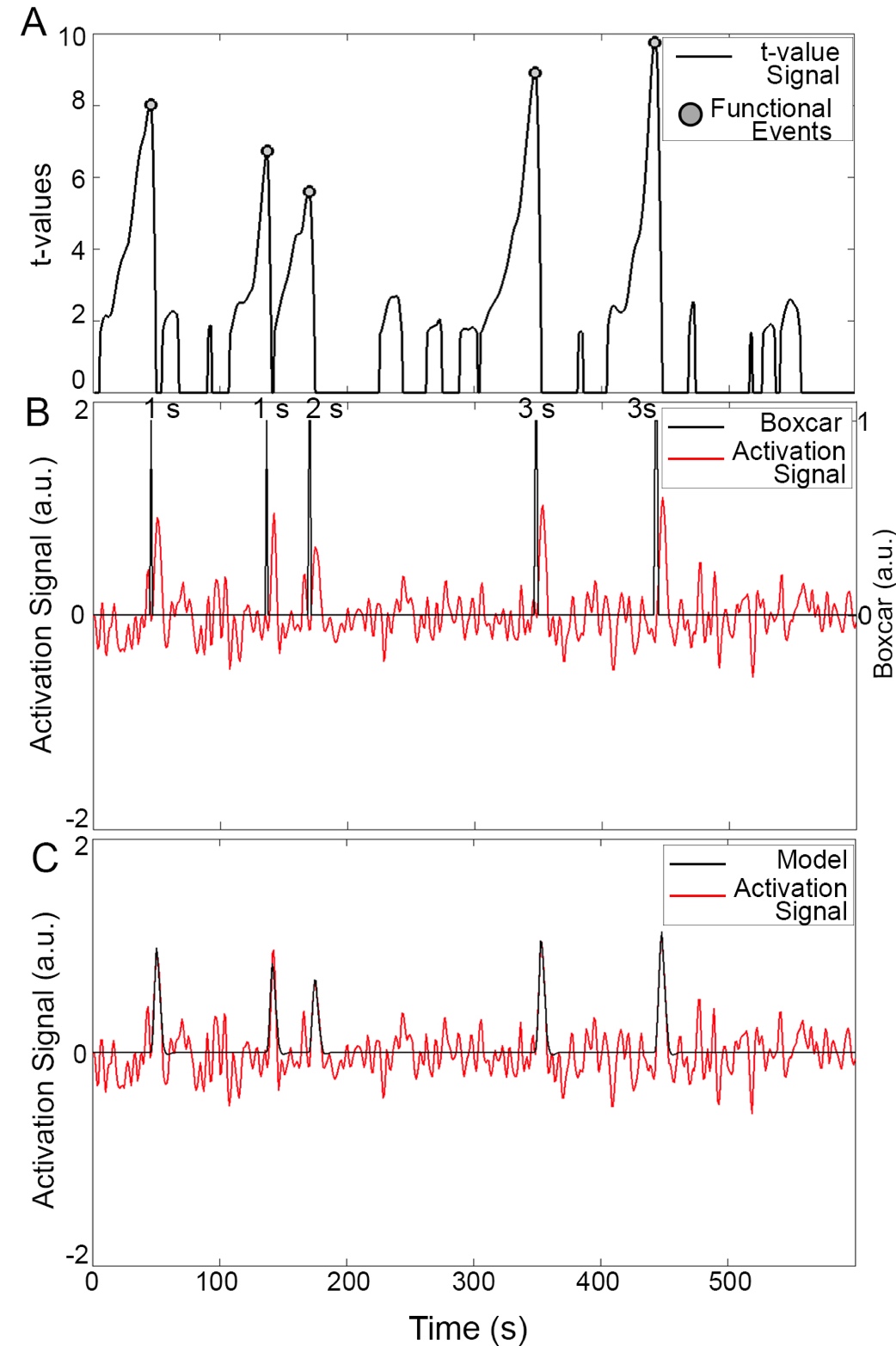
**

**Supplementary Figure 4. Example of t-values signal corresponding to an event-related design synthetic signal with AIDE-identified functional events (A), the corresponding recovered boxcar and durations (B) and model (C) overlapped onto the synthetic CBSI-derived fNIRS activation signal.**

**
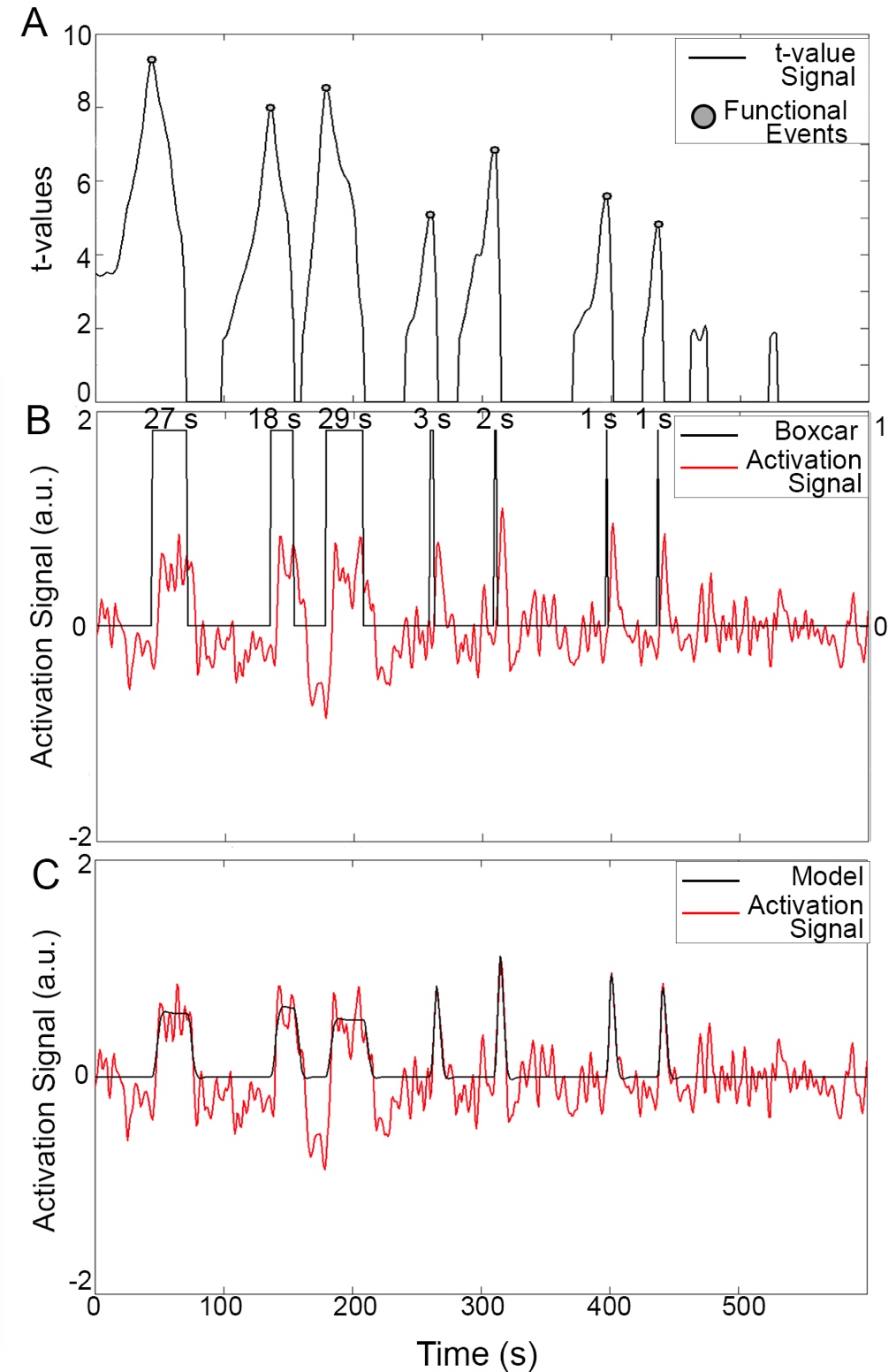
**

**Supplementary Figure 5. Example of t-values signal corresponding to a mixed-design synthetic signal with AIDE-identified functional events (A), the corresponding recovered boxcar and durations (B) and model (C) overlapped onto the synthetic CBSI-derived fNIRS activation signal.**

**3. Lab-based fNIRS Data**

The feasibility of AIDE in the recovery of functional events from real experimental fNIRS data was assessed by applying it to fNIRS data recorded during a conventional block-designed mathematical task described in Section 2.3 of the main text. Within this experiment, prefrontal cortex activity was measured using six measurement channels (see Figure 7 in the main text). Pre-processed HbO_2_ and HHb signals for each channel were combined through the CBSI into six fNIRS activation signals, on which AIDE was applied. AIDE results for a representative participant (P1) are included in Section 3.2 of the main text.

Here, for illustration reasons, we report an example of the t-value signal determined by AIDE to identify functional events for Channel 5, the recovered boxcar and corresponding model (Supplementary Figure 6) for participant P1. More precisely, for this example, AIDE recovered 5/6 events, corresponding to an accuracy of 83.3%. The mean difference between the experimental onsets and the AIDE-identified onset is 1 s with a difference of 7.40±3.44 in terms of their duration (Table 6 in the main text).

**
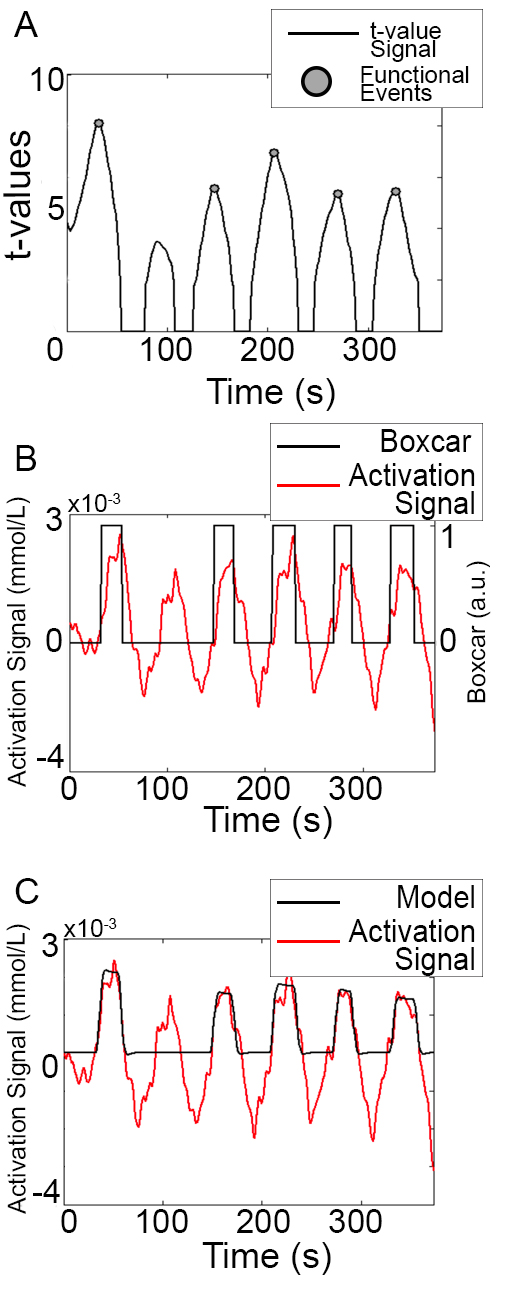
**

**Supplementary Figure 6. Example of (A) t-values signal with AIDE-identified functional events, (B) the resulting boxcar and (C) model for the CBSI-derived Channel 5 fNIRS activation signal.**

**4. AIDE application to additional lab-based fNIRS data**

To further test the feasibility of AIDE in the identification of functional events in real experimental fNIRS data, we applied it to data of another participant for the lab-based mathematical task experiment described in Section 2.3 of the main text.

In this section, we present results referring to this additional participant (healthy female, 20 years old), who underwent the mathematical task described in the main text (Section 2.3). Results are summarized in the following tables. As we did for participant P1, we applied AIDE to the six activation signals created through CBSI. AIDE performance and accuracy was evaluated on the basis of the GLM analysis in the NIRS-SPM software package. More precisely, we identified for each task block whether brain activity occurs or not on the six activation signals by contrasting each task block vs the previous rest phase. Results are shown in Supplementary Table 3 (p<0.008 Bonferroni corrected for multiple comparisons).

|  | **Ch. 1** | **Ch. 2** | **Ch. 3** | **Ch. 4** | **Ch. 5** | **Ch. 6** |
| --- | --- | --- | --- | --- | --- | --- |
| **Contrasts** | **t(75)** | **t(75)** | **t(75)** | **t(75)** | **t(75)** | **t(75)** |
|  |  |  |  |  |  |  |
| **Task#1 vs Rest#1** | 0.90 | 1.17 | 0.91 | 1.65 | 1.76 | 1.71 |
| **Task#2 vs Rest#2** | 3.47 | 4.50 | 5.94 | 4.49 | 4.45 | 4.51 |
| **Task#3 vs Rest#3** | 3.14 | 4.31 | 4.39 | 3.35 | 3.67 | 5.10 |
| **Task#4 vs Rest#4** | -3.96 | -2.60 | -0.82 | -1.19 | -1.14 | 0.42 |
| **Task#5 vs Rest#5** | 1.10 | 1.75 | 1.85 | 2.22 | 2.55 | 3.70 |
| **Task#6 vs Rest#6** | 1.17 | 0.23 | 0.33 | 1.01 | 1.25 | 1.71 |

**Supplementary Table 3.** **Results of the NIRS-SPM GLM analysis for the identification of significant functional activity at single task-block level and t-values for each channel and each contrast.** Significant contrasts are underlined (p<0.008 Bonferroni corrected for multiple comparisons).

Supplementary Table 4 presents the real onset of each task block established in the experimental design and the corresponding onset identified by AIDE using p_thresh_=0.0001.

| Experimental Task Onset (s) | Identified Task  Onset (s) | | | | | |
| --- | --- | --- | --- | --- | --- | --- |
| (s) | **Ch 1** | **Ch 2** | **Ch 3** | **Ch 4** | **Ch 5** | **Ch 6** |
| 31 | 28 | 29 | 28 | 32 | 31 | 31 |
| 89 | 87 | 87 | 88 | 89 | 89 | 90 |
| 157 | * | 155 | 157 | 156 | 156 | 156 |
| 229 | 205 | * | * | * | * | 220 |
| 299 | 300 | 299 | * | 301 | 301 | 302 |
| 358 | * | * | * | * | * | * |

**Supplementary** **Table 4.** **Results of the performance of the AIDE algorithm in the lab-based block design experiment.** The asterisks mark the identified onsets excluded from further analyses as they did not result in significant values for the assigned p_thresh_. Results are FDR corrected for multiple comparisons.

Supplementary Table 5 presents the duration of each task block established in the experimental design and the one identified by AIDE.

| Experimental Task Duration (s) | Identified Task  Duration (s) | | | | | |
| --- | --- | --- | --- | --- | --- | --- |
| (s) | **Ch 1** | **Ch 2** | **Ch 3** | **Ch 4** | **Ch 5** | **Ch 6** |
| 28 | 19 | 16 | 18 | 14 | 15 | 14 |
| 38 | 22 | 24 | 24 | 22 | 22 | 21 |
| 42 | * | 23 | 23 | 12 | 12 | 22 |
| 40 | 21 | * | * | * | * | 27 |
| 28 | 21 | 22 | * | 17 | 20 | 22 |
| 41 | * | * | * | * | * | * |

**Supplementary** **Table 5.** **Duration of the task blocks identified by AIDE.** The asterisk marks the identified onset excluded from further analyses as it did not result in significance at the assigned p_thresh_. Results are FDR corrected for multiple comparisons.

Mean onset and duration differences between the experimental design and the AIDE-identified events are reported in Supplementary Table 6.

|  | Ch 1 | Ch 2 | Ch 3 | Ch 4 | Ch 5 | Ch 6 |
| --- | --- | --- | --- | --- | --- | --- |
| Mean Onset  Difference (s) | 6.00±10.12 | 1.20±1.10 | 0.80±1.30 | 0.80±0.84 | 0.60±0.89 | 2.80±3.63 |
| Mean Duration  Difference (s) | 12.75±5.68 | 10.67±4.16 | 8.00±7.21 | 13.00±2.45 | 9.25±6.99 | 12.00±4.18 |

**Supplementary** **Table 6. Mean ± Standard deviation of differences between the a-priori experimental onsets and durations and AIDE-identified onsets and durations.**

AIDE identified 2/2 task onsets for Channels 2, 3 and 4 and 3/3 task onsets for Channels 5 and 6 corresponding to an accuracy (number of identified onsets/number of real onsets) of 100%, and 1/2 events (Accuracy=50%) were identified for Channel 1. However, AIDE recovered other functional event onsets that did not result significant to the NIRS-SPM single-task analysis (Supplementary Table 4), further suggesting that AIDE improves the performance of GLM-based analyses by enhancing the fit between the fNIRS experimental data and the model. To prove that, we evaluated the performance of the NIRS-SPM GLM analysis using the a-priori onsets established in the experimental design and the corresponding AIDE-identified onsets, contrasting the task blocks regressor versus the rest periods regressor. Results are shown in Supplementary Table 7.

|  | **Ch. 1** | | **Ch. 2** | **Ch. 3** | **Ch. 4** | **Ch. 5** | **Ch. 6** |
| --- | --- | --- | --- | --- | --- | --- | --- |
| **Onsets** | | **t(75)** | **t(75)** | **t(75)** | **t(75)** | **t(75)** | **t(75)** |
| **A-priori onsets** | | 1.67 | 2.94 | 4.17 | 4.10 | 4.43 | 6.27 |
| **AIDE onsets** | | 7.32 | 7.27 | 7.74 | 8.21 | 8.01 | 9.20 |

**Supplementary** **Table 7. Comparison between the GLM analysis performed using the a-priori and the AIDE-identified onsets.** t-values are reported for each channel.

In all the channels, the GLM analysis is improved using the onsets identified through AIDE, further demonstrating that it improves the fit between fNIRS signals and the model.

Supplementary Figure 7 shows an example of the t-value signal recovered by AIDE to identify functional events for Channel 3 of this participant, the recovered boxcar and corresponding model.

**
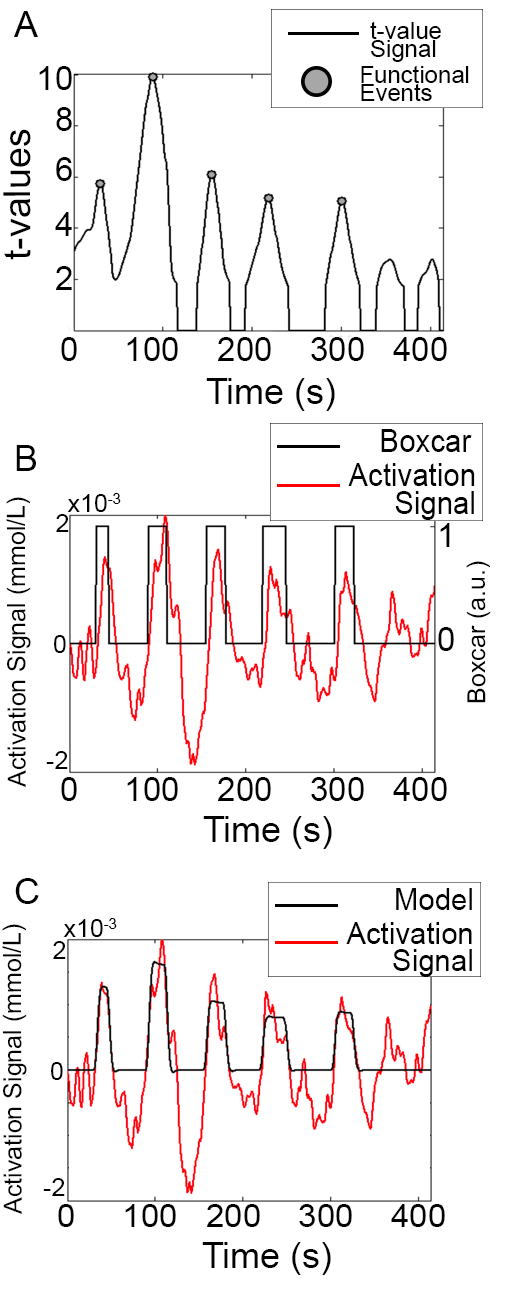
**

**Supplementary Figure 7. Example of (A) t-values signal with AIDE-identified functional events, (B) the resulting boxcar and (C) model for the CBSI-derived Channel 6 fNIRS activation signal.**

**5. Real-world fNIRS data**

In order to demonstrate the feasibility of AIDE in the identification of functional events in real-world fNIRS neuroimaging data, we applied it to a case study (P2) from a real-world fNIRS data recorded during a naturalistic prospective memory (PM) experiment conducted outside the laboratory (see Section 2.4 of the main text). Briefly, the experiment was composed of an uncontaminated and a contaminated ongoing condition (OG and OGc respectively), where the participants was asked to count the number of certain items within the experimental area; two PM conditions, a social and a non-social one (sPM and nsPM respectively), were included as well, in which the participant had to perform the OG task while responding to social and non-social PM targets. Prefrontal cortex hemodynamics was monitored using a 16-channels wireless fNIRS device and heart rate and breathing rate changes were measured through a wearable monitoring belt. AIDE was then applied to the 16 activation signals for the four conditions obtained by CBSI-combining the 16 pre-processed HbO_2_ and HHb data.

In the main text (Section 3.3), we reported results for the sPM condition, showing the locations of the functional events identified by AIDE within the experimental area and the brain maps of the channels resulting involved or not involved each identified event (Figure 9 in the main text). In this section, we report results referring to the OG, nsPM and OGc conditions. In addition, we provide, for each of the four conditions, examples of the t-value signal recovered by AIDE to identify functional events, the recovered boxcar and corresponding model. For illustration reasons, we include examples of two channels for each condition, one for which AIDE identified no events or only one and one for which AIDE recovered more than one event.

**5.1 Ongoing Condition**

Supplementary Figure 8 presents the positions of the AIDE-identified events within the experimental area for the OG condition. Binary brain maps show the channels involved (red circles) and not involved (blue circles) for each identified events. Heart rate and breathing rate signals are provided as well, together with the identified event (red mark). In Supplementary Figure 9, t-values distribution maps across the 16 channels are provided instead of the binary brain maps. White circles mark the channels involved for each identified event.

For this condition, AIDE found a major involvement of the medial channels, and an equal contribution of right and left channels (see Figure 10 in the main text) related to the execution of OG task activities.

**
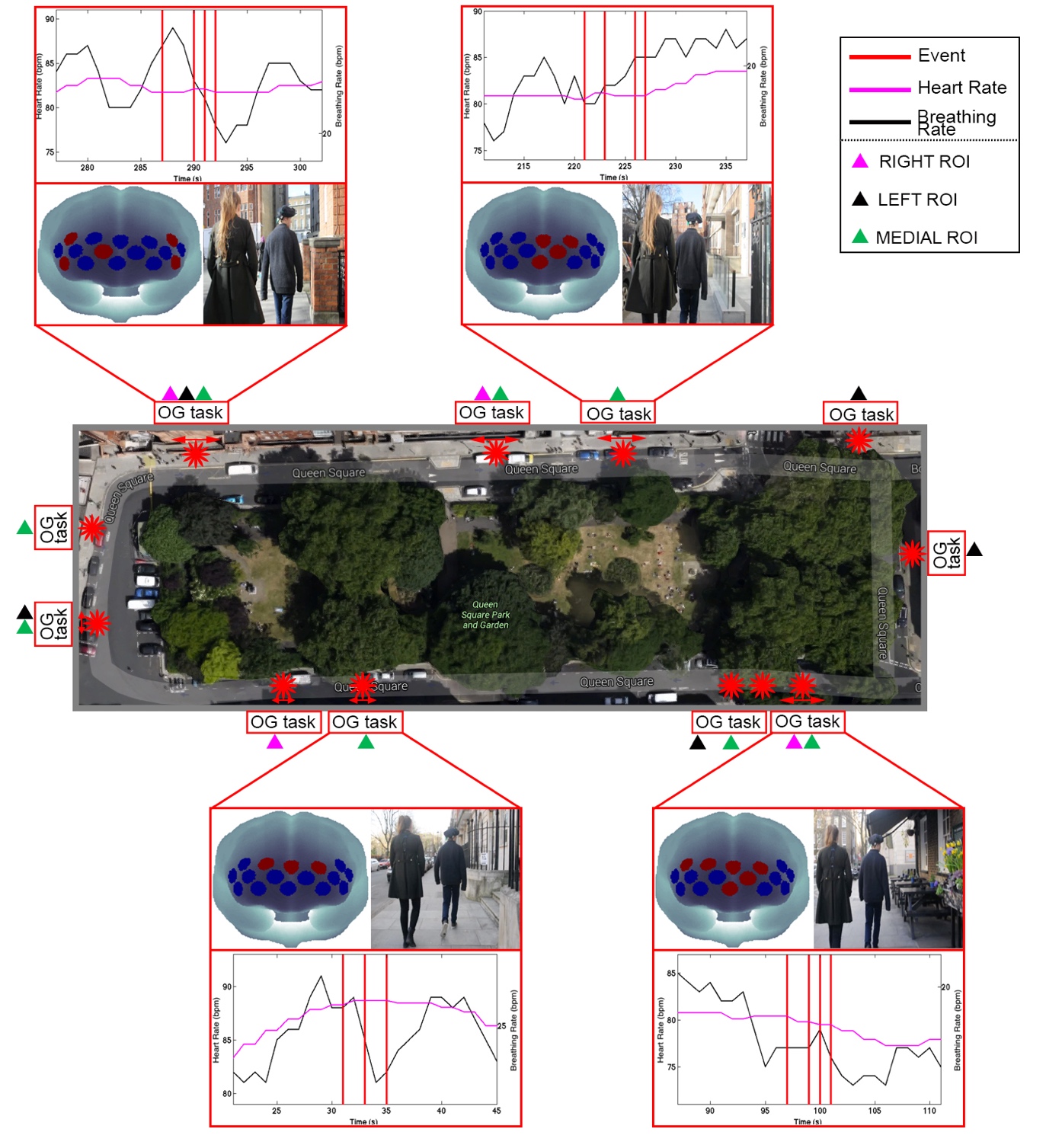
**

**Supplementary Figure 8. Results of the application of the AIDE algorithm to P2 OG condition**. The identified functional events are identified by red asterisks. Functional events are corresponded with participant’s behaviour and the involved ROIs are reported as well as binary brain maps showing the specific responding channels (red circles) and the non-involved channels (blue circles). Functional events are marked with red lines on the heart and breathing rate signals.

**
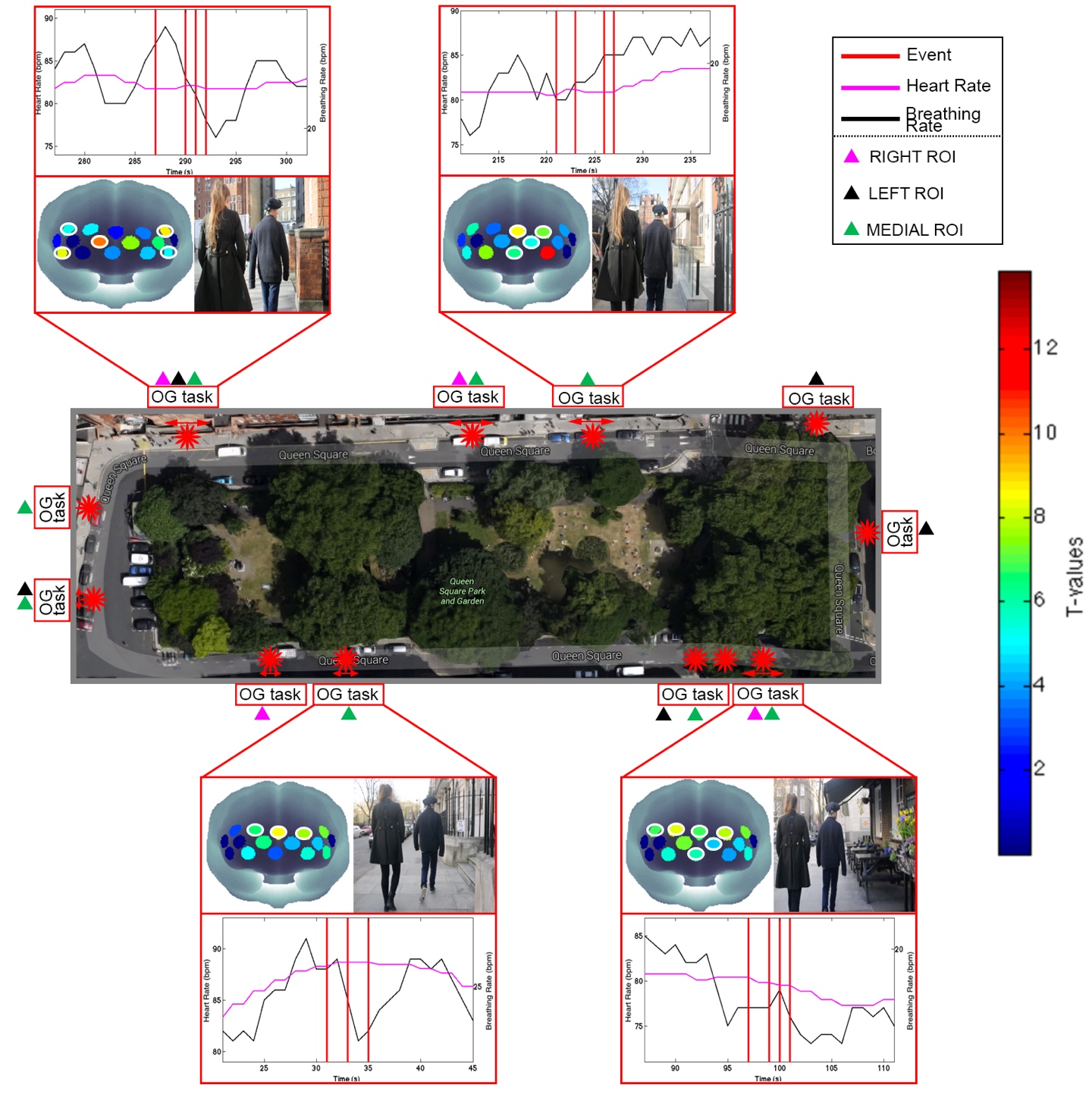
**

**Supplementary Figure 9. Results of the application of the AIDE algorithm to P2 OG condition.** The detected functional events are identified by red asterisks. Functional events are corresponded with participant’s behaviour and the involved ROIs are reported as well as brain maps showing the t-values distribution across the channels activation signals. For each brain map, the channels involved for each specific functional event are indicated by white circles. Functional events are marked with red lines on the heart and breathing rate signals.

Supplementary Figure 10 shows examples of the t-value signals identified by AIDE to detect functional events, the recovered boxcar and corresponding model for Channel 7 and Channel 8 for which AIDE identified 2 events and 4 events respectively.

**
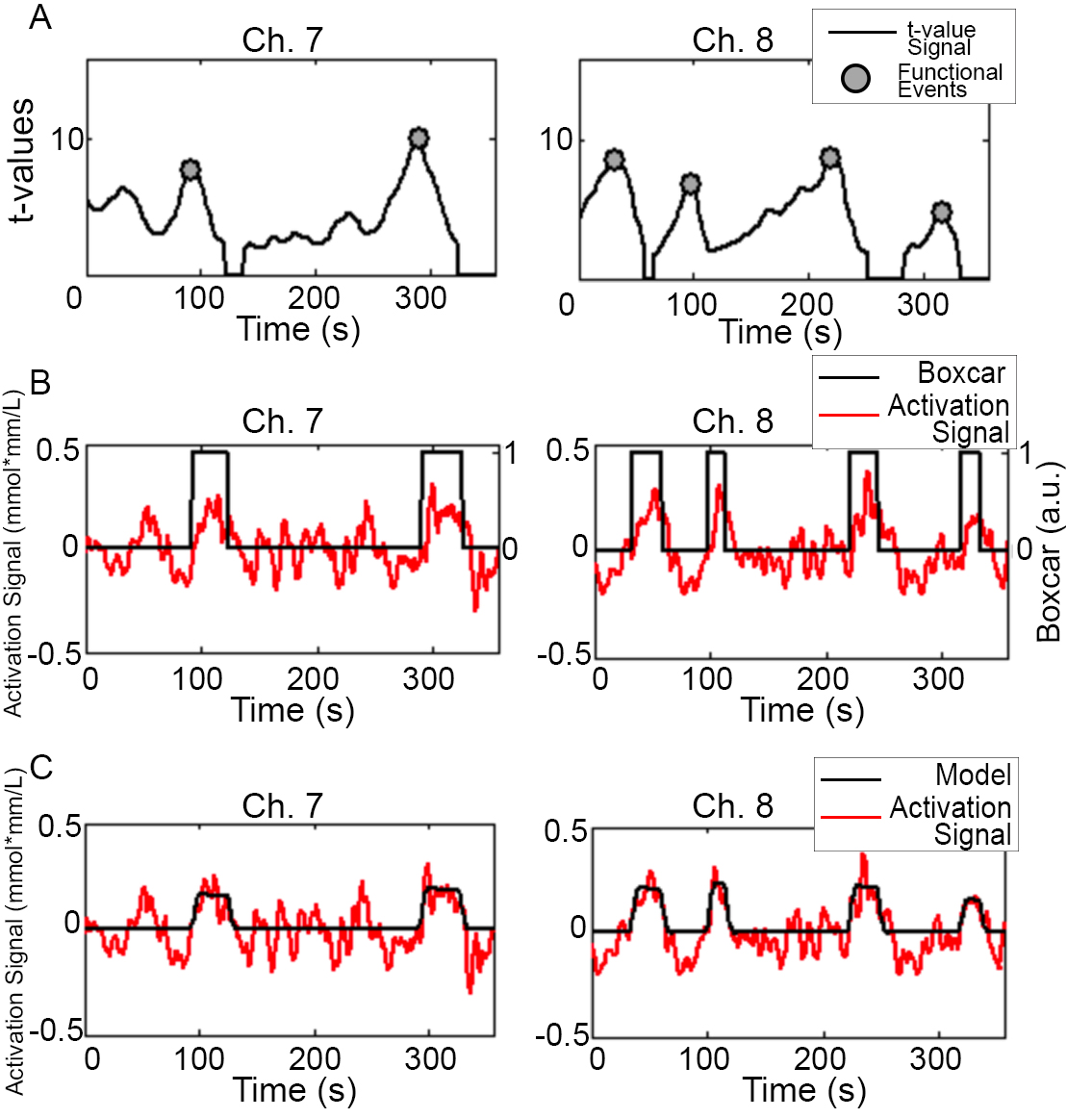
**

**Supplementary Figure 10. Example of (A) t-values signal with AIDE-identified functional events, (B) the resulting boxcar and (C) model for the CBSI-derived Channel 7 and Channel 8 fNIRS activation signals for the OG condition.**

**5.2 Social PM Condition**

Figure 9 in the main text shows the results of the application of AIDE to the sPM condition. In Supplementary Figure 11, we present the same results, but with the t-values distribution maps across the 16 channels instead of the binary brain maps. White circles mark the channels involved for each identified event. Heart rate and breathing rate signals are included as well, together with the identified event (red mark).

For the sPM condition, AIDE recovered the onsets of 3/4 social PM targets, with a corresponding to an accuracy of 75%. In addition, AIDE recovered other events corresponding to OG task activities and road crossing. For the reaching of the social PM targets, there was a major involvement of medial channels, and of the left and right channels in a lesser extent; for the OG activities, there was still a major recruitment of medial channels. Activity-based PM activities (road crossing) were identified as well, with an equal recruitment of all channels. Results of this condition suggest that brain activity to the sPM task occurs when the participant first notices the social PM targets, rather than when he approaches toward them.

**
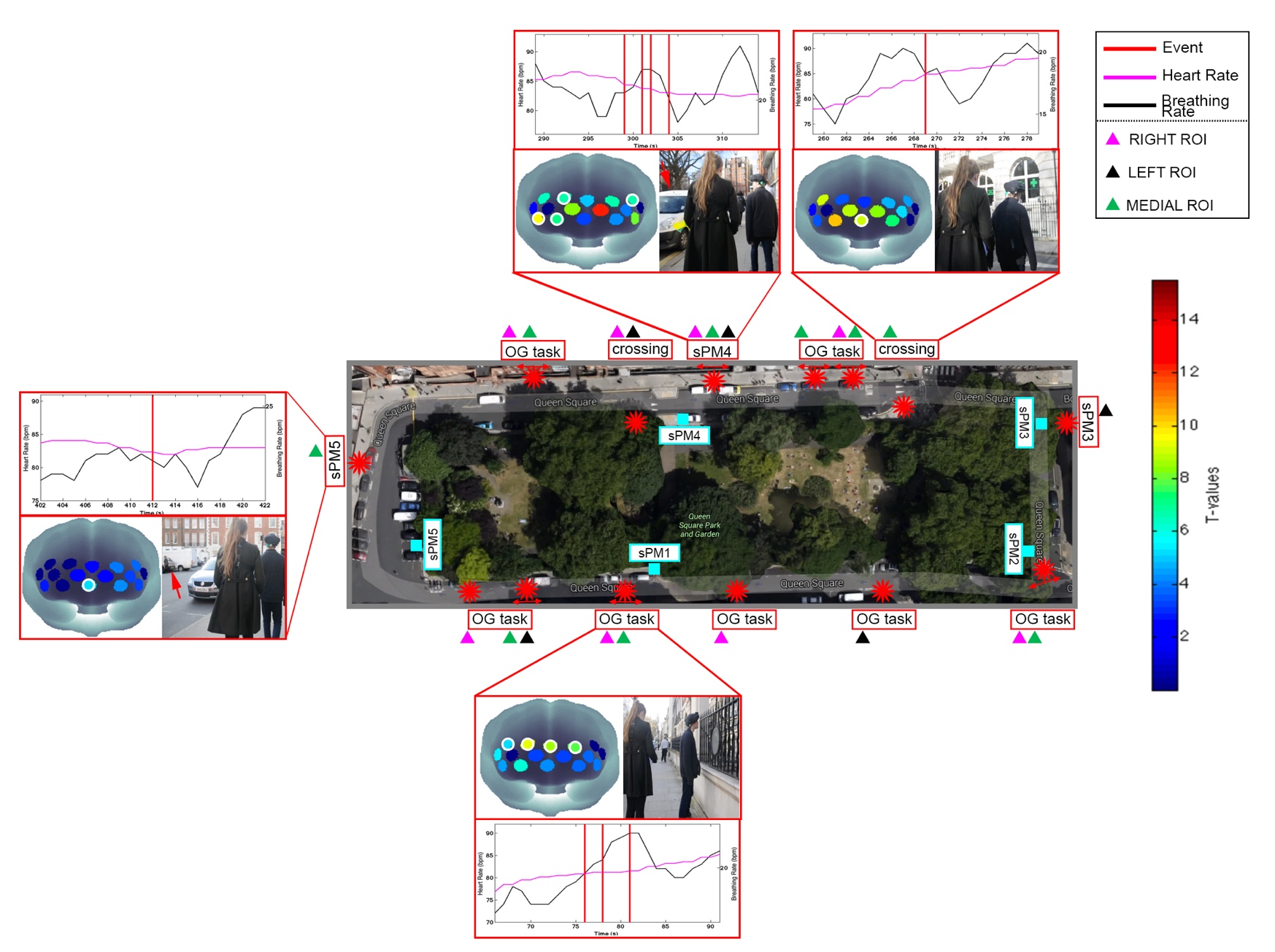
**

**Supplementary Figure 11. Results of the application of the AIDE algorithm to P2 sPM condition.** The detected functional events are identified by red asterisks. Positions of the sPM targets within the square are represented by light blue squares. Functional events are corresponded with participant’s behaviour and the involved ROIs are reported as well as brain maps showing the t-values distribution across the channels activation signals. For each brain map, the channels involved for each specific functional event are indicated by white circles.

Supplementary Figure 12 presents examples of the t-value signal recovered by AIDE to identify functional events, the recovered boxcar and corresponding model for Channel 12, for which AIDE identified 2 events, and Channel 13, for which AIDE identified no events.


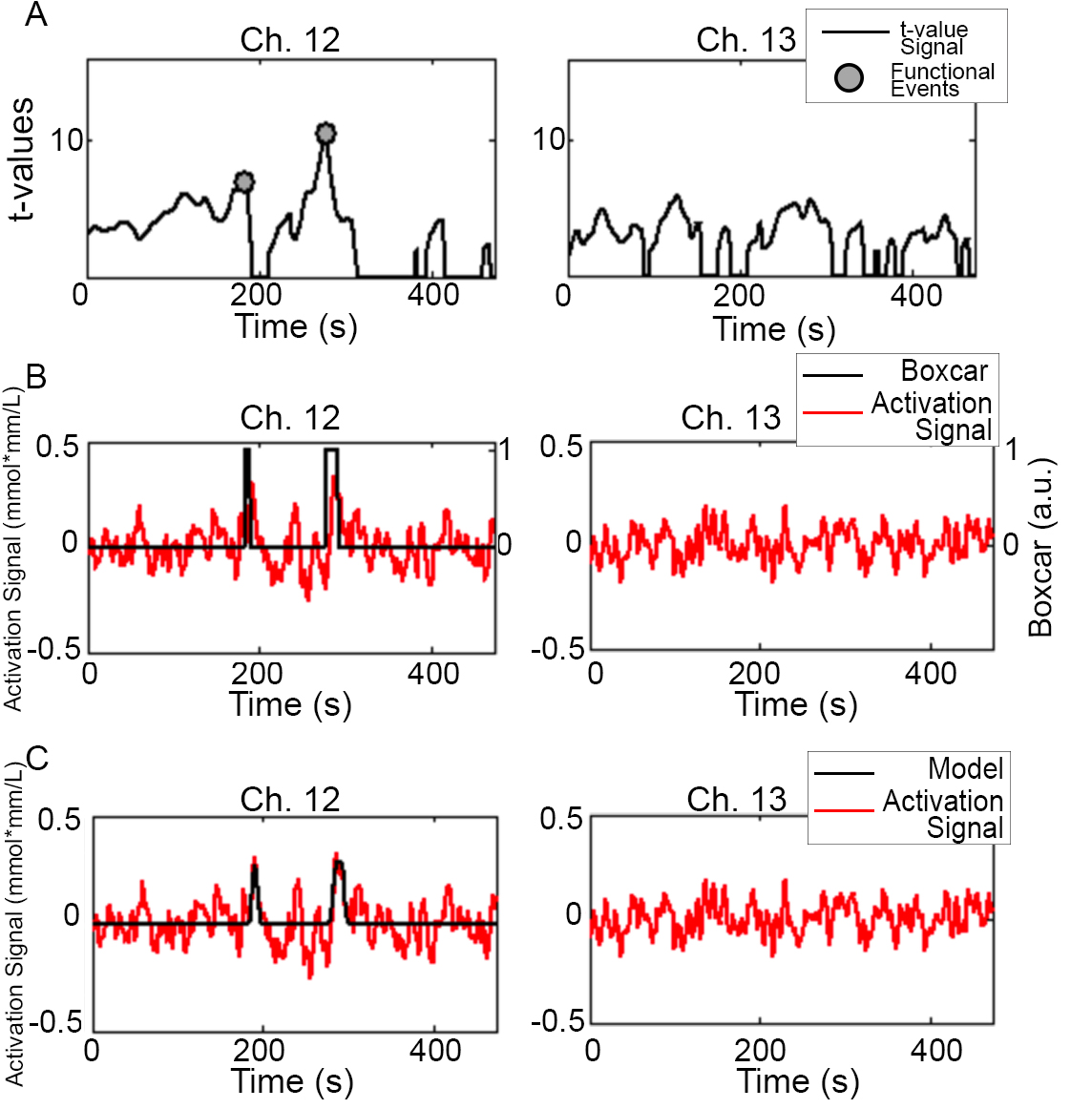


**Supplementary Figure 12. Example of (A) t-values signal with AIDE-identified functional events, (B) the resulting boxcar and (C) model for the CBSI-derived Channel 12 and Channel 13 fNIRS activation signals for the sPM condition.**

**5.3 Non-Social PM Condition**

Supplementary Figure 13 shows the location of the AIDE-identified events within the experimental area for the nsPM condition. Binary brain maps illustrate the channels involved (red circles) and not involved (blue circles) for each identified events. Heart rate and breathing rate signals are provided as well, together with the identified event (red mark). In Supplementary Figure 14, t-values distribution maps across the 16 channels are provided instead of the binary brain maps. White circles mark the channels involved for each identified event.

For the nsPM condition, AIDE identified the onsets of 3/6 non-social PM targets, corresponding to an accuracy of 50%. In addition, AIDE recovered events related to the fulfilment of OG task activities. For the processing of the non-social PM targets, there was a major recruitment of medial channels, and of the left and right channels in a lesser extent; for the OG activities, there was still a major involvement of medial channels. Activity-based PM activities (road crossing) were identified as well, with an equal recruitment of all channels. These results also suggest that functional events are more likely to occur the participant spots the non-social PM targets, rather than when he reaches them.


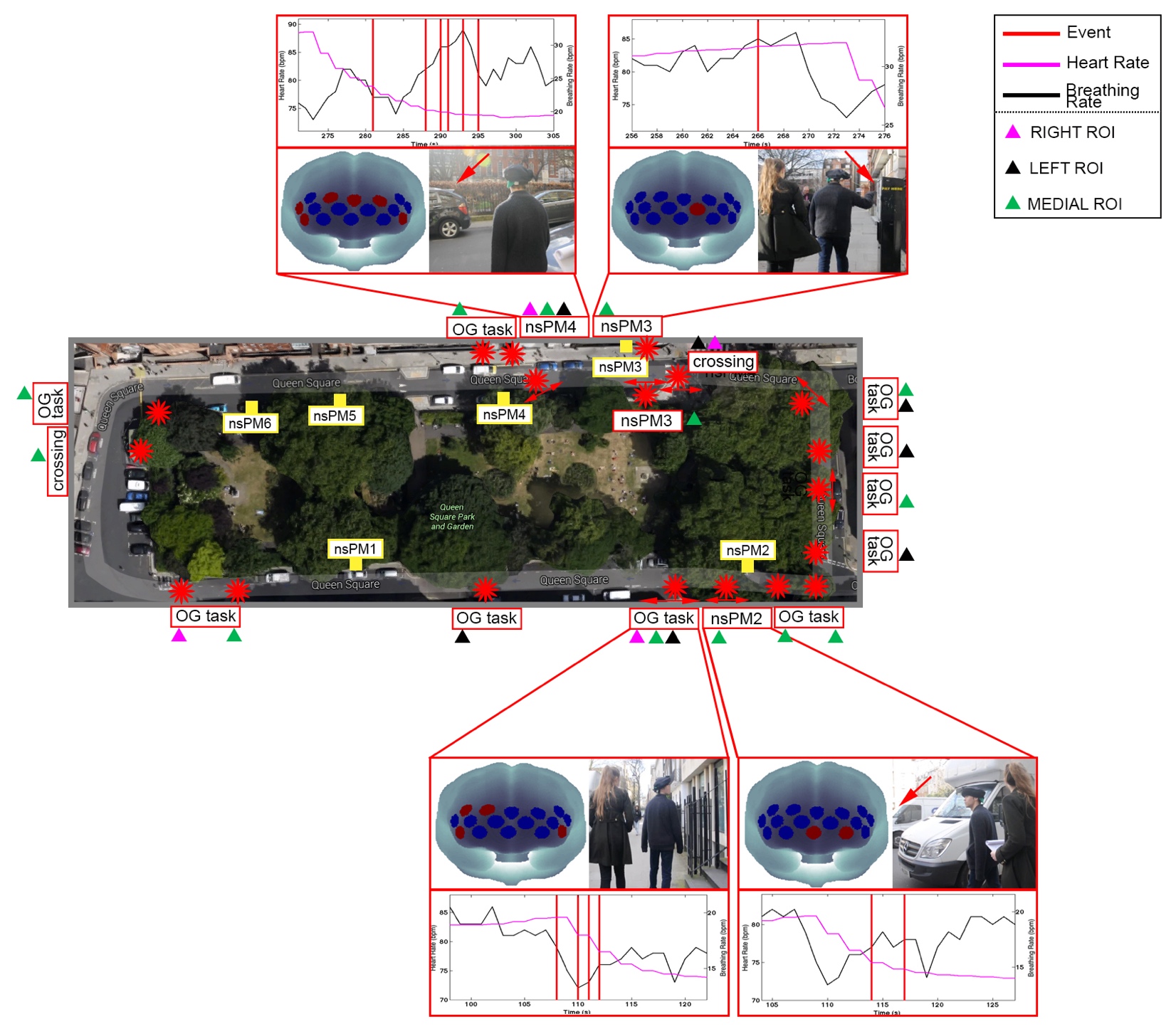


**Supplementary Figure 13. Results of the application of the AIDE algorithm to P2 nsPM condition.** Positions of the nsPM targets within the square are represented by yellow squares. The detected functional events are identified by red asterisks. Functional events are corresponded with participant’s behaviour and the involved ROIs are reported as well as brain maps showing the specific responding channels (red circles) and the non-involved channels (blue circles). Functional events are marked with red lines on the heart and breathing rate signals.

**
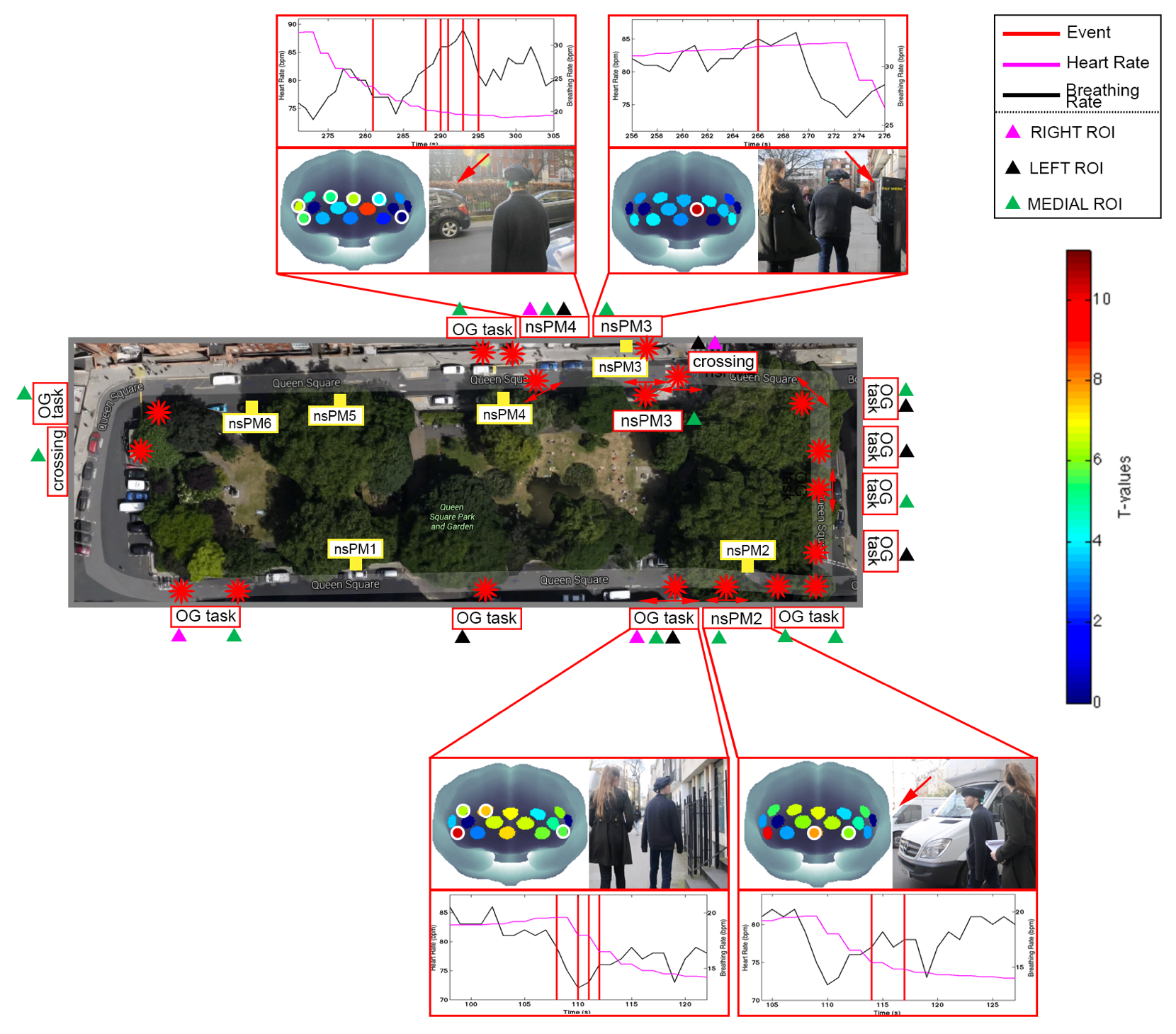
**

**Supplementary Figure 14. Results of the application of the AIDE algorithm to P2 nsPM condition.** The detected functional events are identified by red asterisks. Positions of the nsPM targets within the square are represented by light blue squares. Functional events are matched with participant’s behaviour and the involved ROIs are reported as well as brain maps showing the t-values distribution across the channels activation signals. For each brain map, the channels involved for each specific functional event are indicated by white circles.

Supplementary Figure 15 provides examples of the t-value signals recovered by AIDE to identify functional events, the recovered boxcar and corresponding model for Channel 13, for which AIDE identified no events, and Channel 15, for which AIDE identified 4 events.

**
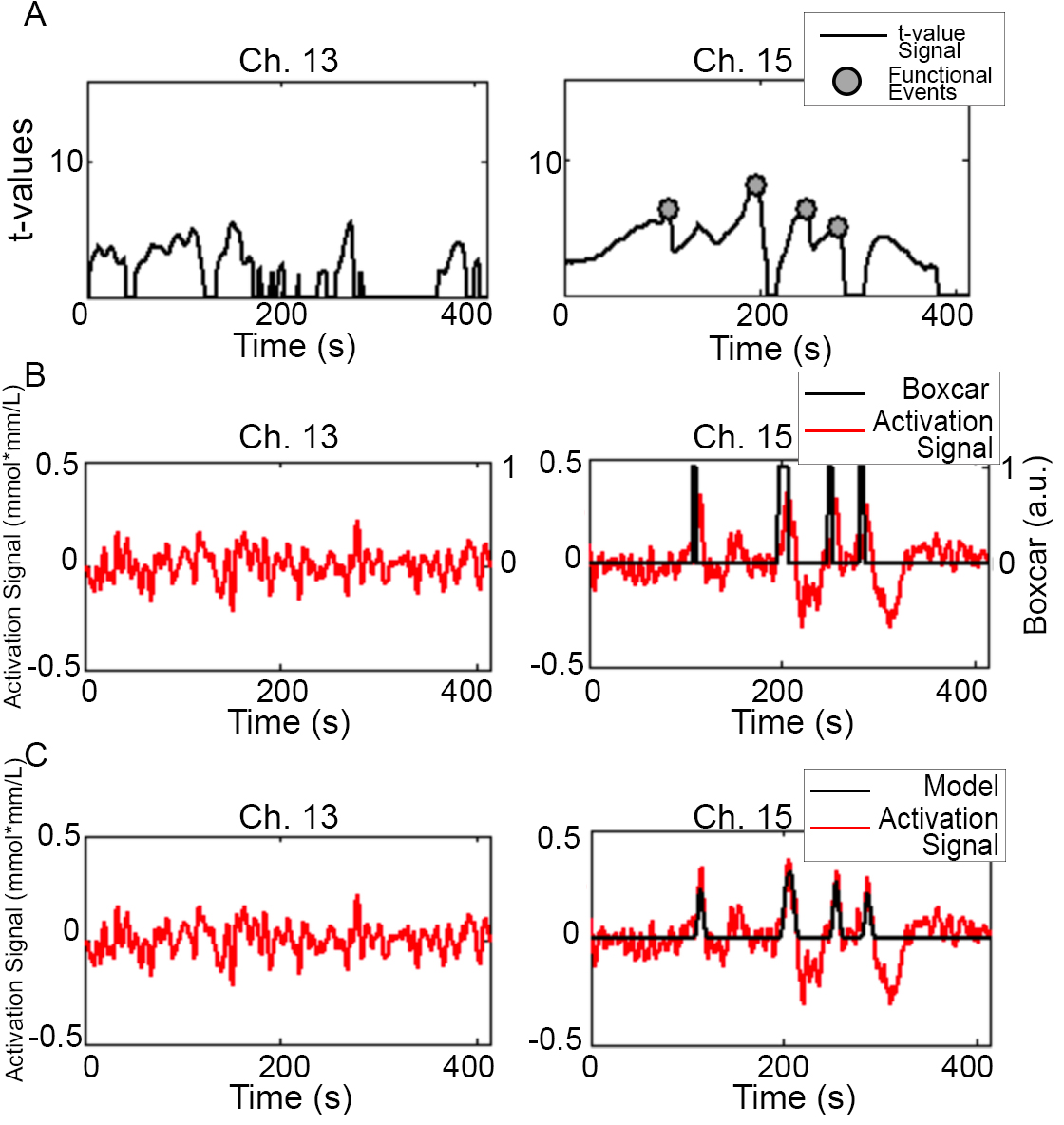
**

**Supplementary Figure 15. Example of (A) t-values signal with AIDE-identified functional events, (B) the resulting boxcar and (C) model for the CBSI-derived Channel 13 and Channel 15 fNIRS activation signals for the nsPM condition.**

**5.4 Contaminated Ongoing Condition**

Supplementary Figure 16 illustrates the location of the AIDE-identified events within the experimental area for the OGc condition. Binary brain maps show the channels involved (red circles) and not involved (blue circles) for each identified events. Heart rate and breathing rate signals are provided as well, together with the identified event (red mark). In Supplementary Figure 17, t-values distribution maps across the 16 channels are provided instead of the binary brain maps. White circles mark the channels involved for each identified event.

For the execution of the OGc task, AIDE identified a major involvement of the medial channels (see Figure 8 in the main text) in the execution of the OG task.

**
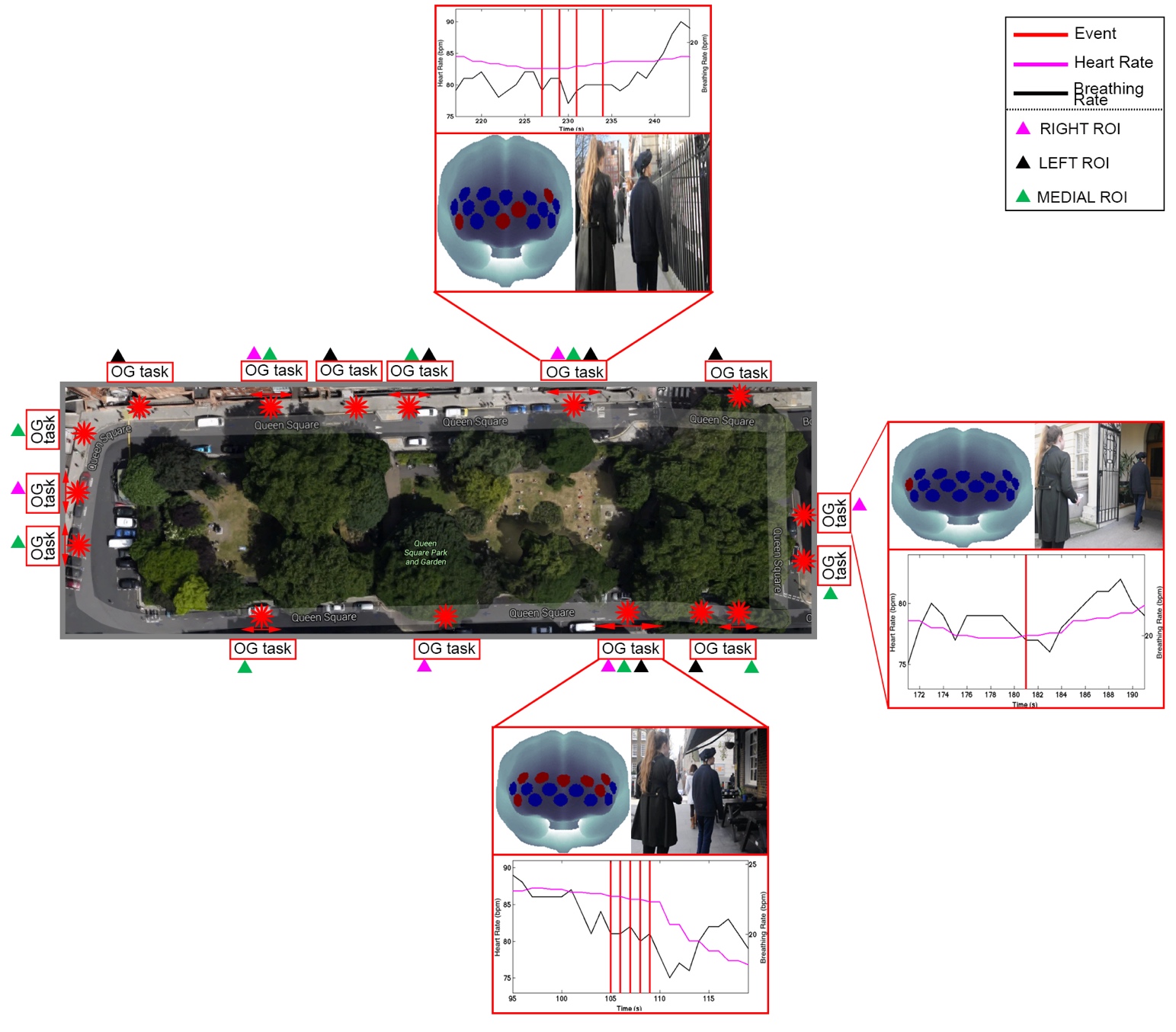
**

**Supplementary Figure 16. Results of the application of the AIDE algorithm to P2 OGc condition**. The identified functional events are identified by red asterisks. Functional events are corresponded with participant’s behaviour and the involved ROIs are reported as well as binary brain maps showing the specific responding channels (red circles) and the non-involved channels (blue circles). Functional events are marked with red lines on the heart and breathing rate signals.

**
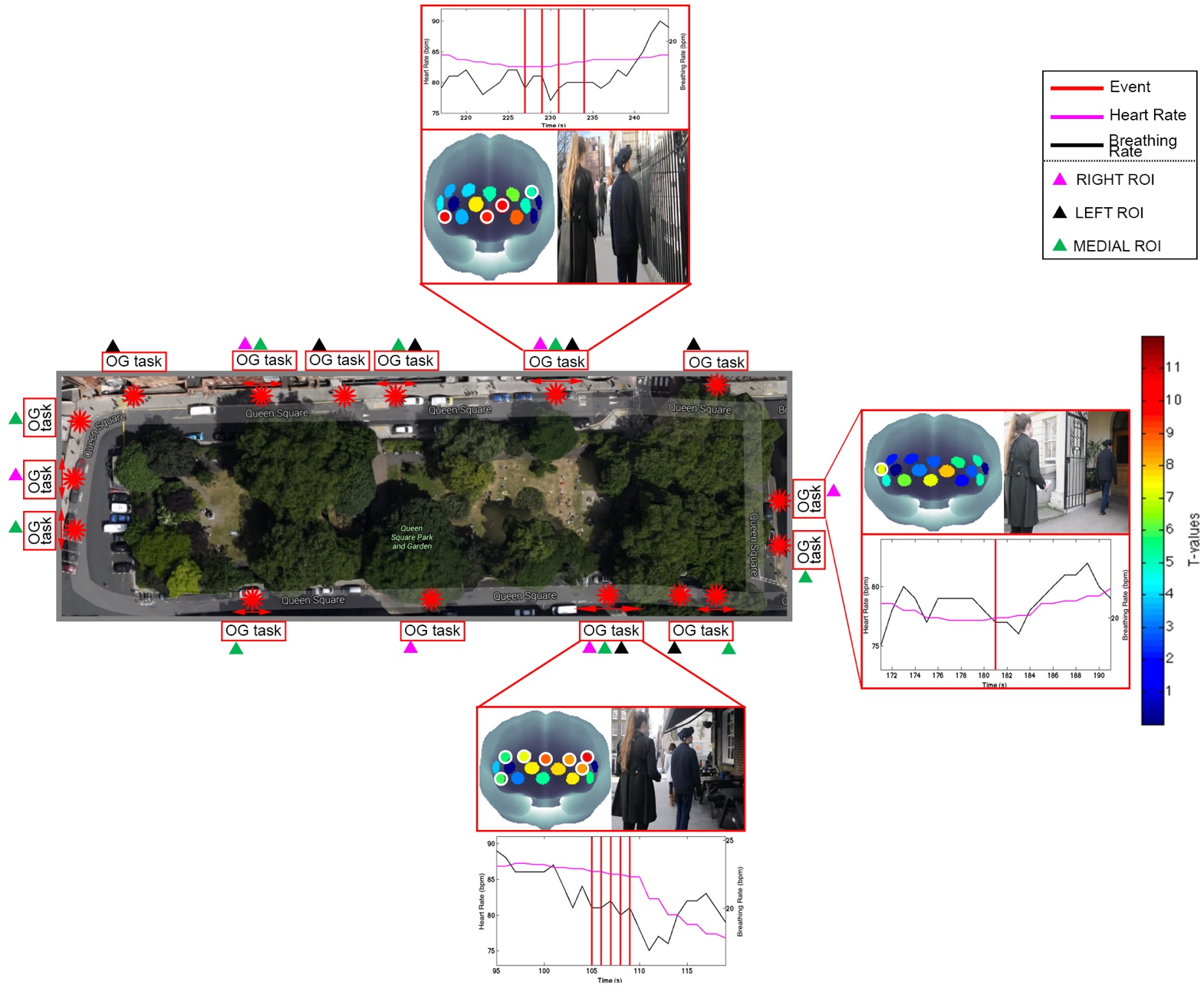
**

**Supplementary Figure 17. Results of the application of the AIDE algorithm to P2 OG condition.** The detected functional events are identified by red asterisks. Functional events are corresponded with participant’s behaviour and the involved ROIs are reported as well as brain maps showing the t-values distribution across the channels activation signals. For each brain map, the channels involved for each specific functional event are indicated by white circles. Functional

Supplementary Figure 18 shows examples of the t-value signals recovered by AIDE to identify functional events, the recovered boxcar and corresponding model for Channel 1, for which AIDE identified 2 events, and Channel 9, for which AIDE identified 3 events.


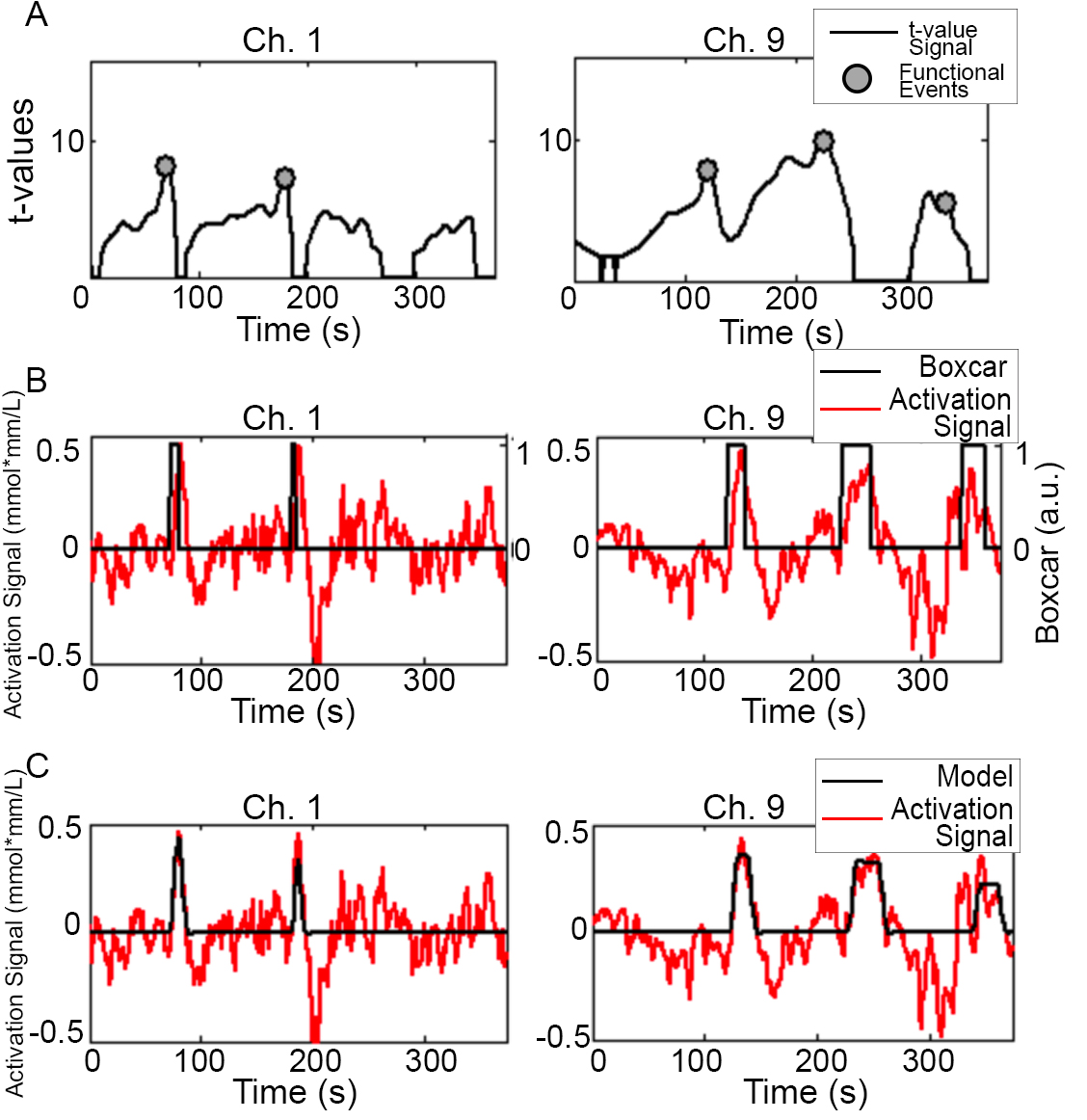


**Supplementary Figure 18. Example of (A) t-values signal with AIDE-identified functional events, (B) the resulting boxcar and (C) model for the CBSI-derived Channel 1 and Channel 9 fNIRS activation signals for the OGc condition.**

**5.5 AIDE-identified onsets and durations**

Supplementary Table 8 summarizes the results obtained by applying AIDE to P2 real-world fNIRS data. More precisely, the identified onsets and corresponding durations are reported for each channel and for each condition. For this participant, the maximum number of events identified per channel is 4 and the minimum is 1; the mean identified duration across the four conditions is 16.8 s, with a maximum of 49 s and a minimum of 1 s.

|  | **OG** | | **sPM** | | **nsPM** | | **OG c** | |
| --- | --- | --- | --- | --- | --- | --- | --- | --- |
|  | **Onsets (s)** | **Durations (s)** | **Onsets (s)** | **Durations (s)** | **Onsets (s)** | **Durations (s)** | **Onsets (s)** | **Durations (s)** |
| **Ch. 1** | 14 | 7 | 94 | 8 | 244 | 9 | 72 | 8 |
|  |  |  | 326 | 2 | 291 | 8 | 181 | 4 |
|  |  |  | 384 | 6 |  |  |  |  |
| **Ch. 2** | 101 | 6 | 78 | 17 | 110 | 19 | 108 | 24 |
|  | 237 | 12 | 180 | 4 |  |  | 268 | 4 |
|  | 292 | 19 | 282 | 27 |  |  | 345 | 12 |
| **Ch. 3** | 13 | 8 | 9 | 13 | 3 | 11 | 107 | 9 |
|  | 290 | 39 | 302 | 19 | 112 | 16 | 234 | 1 |
|  |  |  |  |  | 293 | 4 |  |  |
| **Ch. 5** | 31 | 26 | 76 | 15 | 111 | 19 | 35 | 10 |
|  | 100 | 12 | 304 | 1 | 288 | 13 | 108 | 20 |
|  | 318 | 12 |  |  |  |  | 170 | 3 |
|  |  |  |  |  |  |  | 350 | 23 |
| **Ch. 6** | 241 | 41 | 299 | 12 | 10 | 33 | 336 | 22 |
|  |  |  |  |  | 191 | 40 |  |  |
|  |  |  |  |  | 273 | 25 |  |  |
|  |  |  |  |  | 351 | 30 |  |  |
| **Ch. 7** | 93 | 31 | 47 | 17 | 140 | 28 | 107 | 27 |
|  | 291 | 35 | 291 | 18 | 215 | 11 | 247 | 12 |
| **Ch. 8** | 33 | 26 | 81 | 10 | 236 | 17 | 36 | 8 |
|  | 99 | 15 | 290 | 18 | 390 | 11 | 106 | 1 |
|  | 221 | 25 |  |  |  |  |  |  |
|  | 317 | 17 |  |  |  |  |  |  |
| **Ch. 9** | 97 | 22 | 269 | 36 | 114 | 17 | 122 | 16 |
|  | 226 | 17 | 412 | 1 | 229 | 11 | 227 | 27 |
|  |  |  |  |  | 361 | 7 | 337 | 22 |
| **Ch. 10** | 99 | 15 | 287 | 44 | 112 | 17 | 231 | 33 |
|  | 227 | 11 | 386 | 24 | 266 | 49 |  |  |
|  | 307 | 23 |  |  |  |  |  |  |
| **Ch. 11** | 35 | 24 | 81 | 9 | 158 | 26 | 108 | 19 |
|  | 100 | 15 | 290 | 18 | 295 | 4 | 248 | 28 |
|  | 223 | 32 |  |  |  |  | 351 | 22 |
|  | 318 | 12 |  |  |  |  |  |  |
| **Ch. 12** | 243 | 14 | 184 | 6 | 117 | 6 | 123 | 26 |
|  | 292 | 14 | 278 | 14 | 189 | 16 | 267 | 13 |
| **Ch. 13** | 89 | 26 |  |  |  |  | 105 | 33 |
|  | 316 | 12 |  |  |  |  | 248 | 13 |
| **Ch. 14** | 99 | 16 | 140 | 20 | 93 | 34 | 109 | 28 |
|  | 165 | 35 | 301 | 7 | 171 | 1 | 229 | 42 |
|  | 291 | 38 |  |  | 218 | 10 | 350 | 16 |
| **Ch. 15** | 183 | 10 | 50 | 10 | 108 | 4 | 117 | 1 |
|  | 287 | 9 | 237 | 7 | 197 | 10 | 201 | 15 |
|  |  |  | 326 | 19 | 248 | 5 | 256 | 2 |
|  |  |  |  |  | 281 | 5 | 326 | 13 |

**Supplementary Table 8. Onsets and corresponding duration of functional events identified through AIDE, for the four conditions of the real-world PM experiment and the analysed channels.**
